# High-resolution palynology reveals the land use history of a Sami *renvall* in northern Sweden

**DOI:** 10.1007/s00334-016-0596-5

**Published:** 2016-11-18

**Authors:** Ilse M. Kamerling, J. Edward Schofield, Kevin J. Edwards, Kjell-Åke Aronsson

**Affiliations:** 1 Department of Geography and Environment, School of Geosciences, University of Aberdeen, Elphinstone Road, Aberdeen, AB24 3UF UK; 2 Department of Archaeology, School of Geosciences, University of Aberdeen, Elphinstone Road, Aberdeen, AB24 3UF UK; 3 Ájtte, Swedish Mountain and Sami Museum, Box 116, 962 23 Jokkmokk, Sweden

**Keywords:** Forest Sami, Boreal forest, Reindeer herding, Pollen analysis, Coprophilous fungal spores

## Abstract

The limited availability of historical and archaeological evidence means that much is still unknown about the development of Sami reindeer herding in Fennoscandia in both the recent and more distant past. To address this problem, high-resolution palynological analyses, ^14^C and ^210^Pb dating were undertaken on two adjacent (<25 m apart) peat profiles collected at a recently abandoned reindeer gathering pen (*renvall*) near Jokkmokk (~66.6°N, 19.8°E) in the boreal forest of northern Sweden. The aim was to assess the impact of Sami reindeer herding on the local environment through a study of pollen, coprophilous fungal spores, microscopic charcoal and sedimentology. The samples collected from within an annex to the *renvall* indicate cycles of use and abandonment of the pen on a multi-decadal timescale between ~ad 1800–2008, most obviously in the coprophilous fungal spore archive. The pattern and timing of these cycles confirm events previously known only from oral histories. Although the local pollen assemblage zones associated with the phasing of activity were reproducible in a second peat core beyond the boundary of the *renvall*, the coprophilous fungal spore signal in this paired profile was much less distinctive, possibly due to the typically shorter dispersal distances for these microfossils in comparison to pollen grains.

**Electronic supplementary material:**

The online version of this article (doi:10.1007/s00334-016-0596-5) contains supplementary material, which is available to authorized users.

## Introduction

**Fig. 1:**
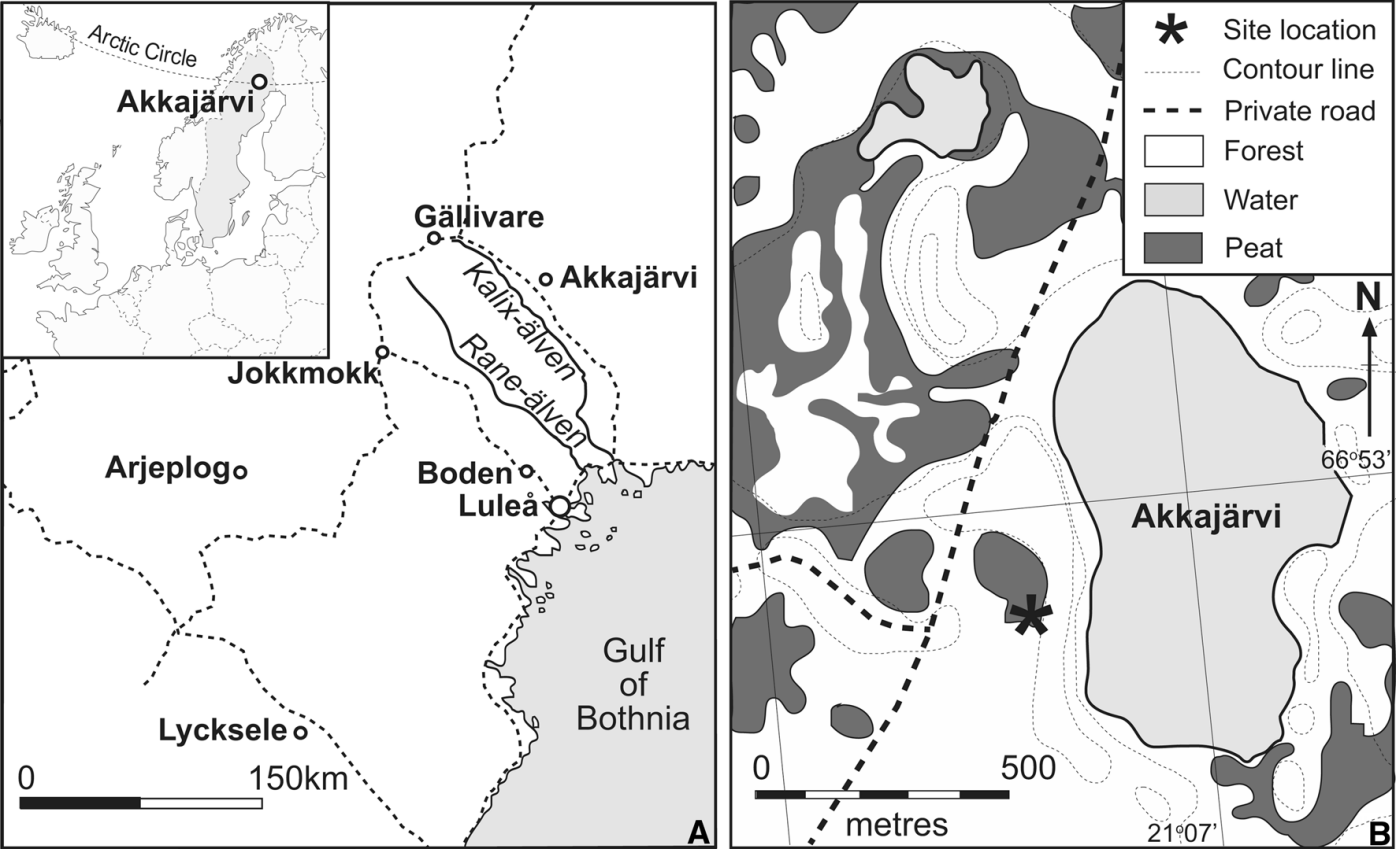
**a** Location of the study site at Akkajärvi and other major towns including the winter market town of Jokkmokk; **b** the location of the *renvall* close to the lake. Contours are at 5 m intervals

This paper compares the oral histories of 20th century forest Sami reindeer herding at a recently abandoned gathering pen (*renvall*) at Akkajärvi, northern Sweden (Fig. 1), with the results of palynological analyses. The aims of this study are:

(i) to assess the response and sensitivity of pollen and various non-pollen palynomorphs (NPPs) to cyclically-recurring reindeer pastoralism at a fixed location in a boreal forest setting;
(ii) to investigate whether patterns revealed in the palaeoecological record match the timing of activity at, and abandonment of the site, as understood from oral histories.

Akkäjarvi featured in an earlier palynological study of forest reindeer herding in Sweden (Aronsson 1991), which showed that such analyses provided a means for detecting and evaluating the landscape response to reindeer pastoralism in northern Fennoscandia. A higher-resolution analysis is presented here, supplemented by an improved site chronology based on the Bayesian modelling of a combined series of ^210^Pb and ^14^C dates (Blaauw and Christen 2011). The analyses of coprophilous fungal spores, microscopic charcoal and loss-on-ignition (LOI) are incorporated along with pollen analysis (Kuoppamaa et al. 2009). Coprophilous fungal spores offer evidence of biotic impact in the form of livestock grazing and gathering (van Geel et al. 2003; Cugny et al. 2010; Baker et al. 2013). Microscopic charcoal is employed as a measure of fire incidence (Patterson et al. 1987; Ericsson et al. 2000; Niklasson and Granström 2000) which may be linked to the use of domestic and smudge fires, the latter being the lingering smoke-producing fires that protect the reindeer from mosquitoes (van Dyke 1901; Aronsson 1991). LOI is used as a proxy for soil erosion (Edwards and Rowntree 1980; Evans 1996).

The Sami form a cultural group that is currently spread across Sápmi, an area covering northern Norway, Sweden, Finland and the Russian Kola Peninsula. Much remains unknown about their cultural history owing to a scarcity of historical and archaeological evidence. Debate continues over the nature and timing of the domestication of *Rangifer tarandus* (reindeer) by the Sami, which is variously placed as early as the end of the last glacial (Weichselian) period (Bogoras 1924; Jochelson 1926), the 1st millennium bc (Helskog 1988), the 1st millennium ad (Östlund and Bergman 2006), ad 800–1000 (the Viking Age) (Manker 1947; Ruong 1982), or as late as the 16th–18th century (Müller-Wille et al. 2006; Bjørnstad et al. 2012). The transition from hunting to herding (semi-nomadic pastoralism) must have resulted in changes in the relationship between people, their animals and the environment (most notably, the vegetation).

The impacts of Sami reindeer hunting on the environment are believed to have been minimal (Aronsson 1991, 1994), but are distinguishable in the palynological record (Hicks 1993; Bergman et al. 2004a; Hörnberg et al. 2005; Josefsson et al. 2009, 2010), much like hunter-fisher-gatherer societies with partially related lifestyles (Edwards 1996; Edwards et al. 2009; but see Woelders et al. 2016). Historical and palaeoecological data place the transition from reindeer hunting to true, intensive reindeer herding around the 17th or 18th century ad (Aronsson 1991; Lundmark 2007). This method involved the regular gathering of small, tame herds of animals for the purposes of milking, calving, calf marking, slaughtering and protection (Aronsson 1991), while the daily subsistence requirements continued to be met through hunting and gathering (Niklasson et al. 1994; Bergman et al. 2004b; Müller-Wille et al. 2006). Reindeer were lured to their summer herding grounds with the help of peat-fuelled smudge fires. In northern Norrland, extensive reindeer herding replaced the intensive method around 1920–1930. Under this system the herds became larger, control over the animals was reduced, and reindeer milk was replaced by that of cows and also goats as people became more settled. Pens previously used for reindeer milking were increasingly used for summer calf-marking.

Four main effects of recurring activity during the 16th to 19th centuries at semi-permanent Sami reindeer herding settlements in the Arjeplog area (~66.1°N 17.9°E; Fig. 1) were proposed by Freschet et al. (2014): (1) organic matter deposition, both by the Sami and their herds; (2) soil compaction through trampling; (3) destruction of the field layer; (4) selective felling of *Pinus sylvestris* for fuel and building purposes. Palynologically, the creation of local openings in the boreal forests of northern Fennoscandia, to provide spaces for dwellings and livestock, is recognizable by a decline in trees (Hicks 1976; Vuorela 1976; Berglund et al. 2008). An increase in Poaceae forms the main response among non-arboreal taxa, but the strength of this signal in the palynological record weakens with increasing distance from the disturbance (Suominen 1975; Aronsson 1991; Hicks 1993; Aronsson 1994).

A set of plants that is indicative of reindeer herding in the boreal forests of northern Sweden has been identified by Aronsson (1991); representative pollen types include Poaceae, *Melampyrum*, *Solidago virgaurea*, *Achillea millefolium*, *Epilobium angustifolium*, *Rumex acetosa/acetosella*, *Chenopodium*, *Silene dioica* and *Urtica*. These taxa respond positively to increased light levels following clearance, are resistant to soil trampling and typically react positively to the increased soil nutrient levels provided by inputs from reindeer dung, domestic waste and ash from smudge fires. Recovery following the abandonment of disturbed areas is characterised by a general secondary succession whereby the Poaceae-dominated vegetation is replaced first by ericaceous heaths and *Betula*, then by *Pinus* and finally by *Picea* (Bradshaw and Zackrisson 1990; Jonsson and Esseen 1998; Freschet et al. 2014). This recovery may take considerably longer than the duration of human impact, grazing activity and the resulting nitrogen enrichment (Walker and Wardle 2014). At reindeer herding settlements near Arjeplog (450–550 m a.s.l.), secondary succession was still underway more than 100 years after abandonment, with the old activity areas forming islands of *B. pubescens* within the *P. sylvestris*-dominated forest (Freschet et al. 2014). Once the vegetation within the *renvall* has recovered, it barely stands out from the surrounding forest, and can be recognised only by larger specimens of trees that had been left to provide shade for the reindeer (Östlund et al. 2003).

## Study site

### Cultural history

**Fig. 2:**
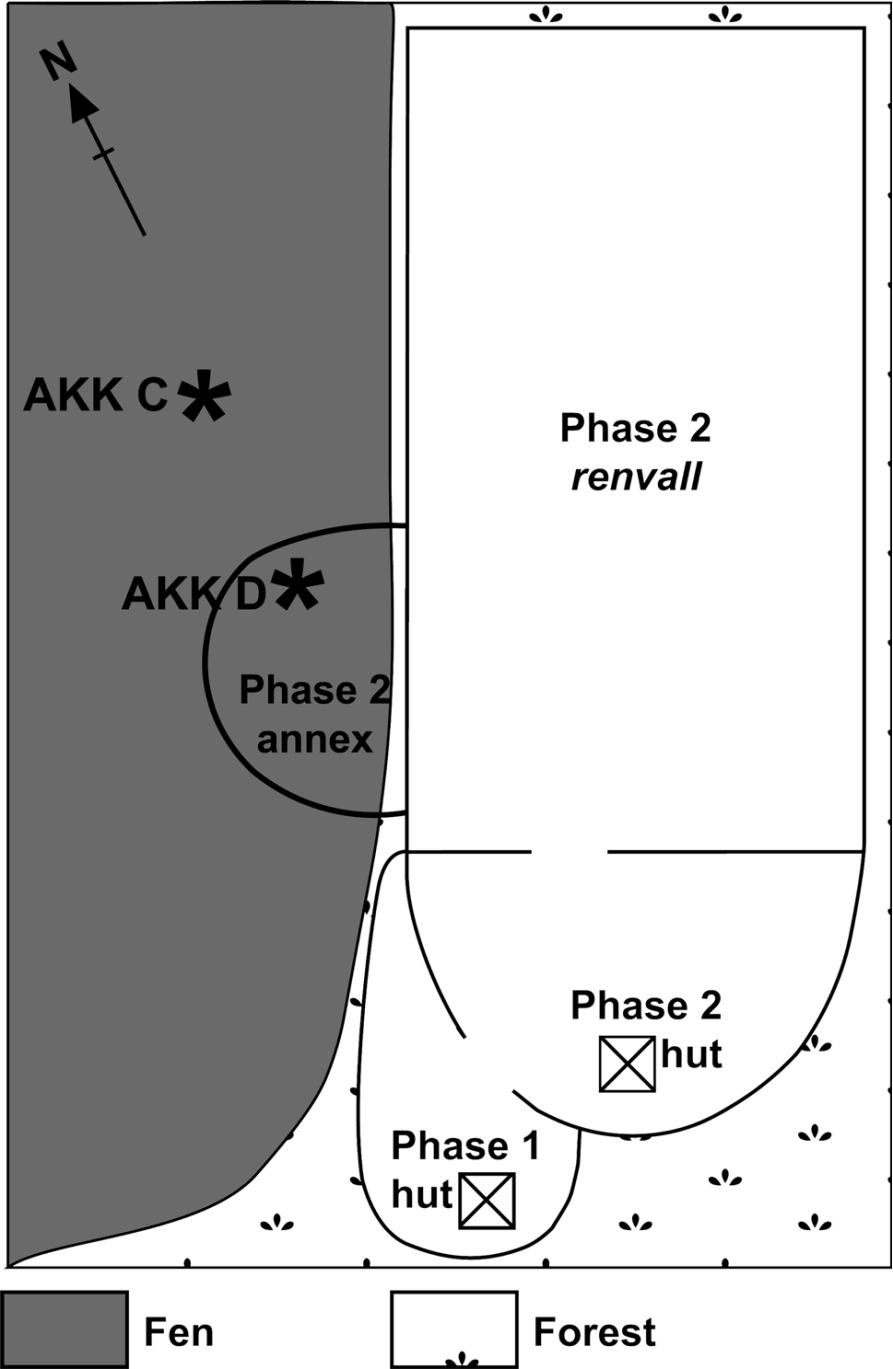
Schematic plan of the layout of the *renvall* at Akkajärvi during the intensive (phase 1; pre-*c.* 1920–1930) and extensive (phase 2; ~1970–1990) reindeer herding periods. Sampling locations AKK D (inside the annex) and AKK C (on the fen) are depicted as stars. The image is not to scale

Akkajärvi is the Finnish translation of the old Sami name Akkajaure (*Akka* referring to a female divinity, and *jaure*/*järvi* meaning lake). This name was given to a lake located ~60 km northeast of Jokkmokk (Fig. 1b) and is here also used to identify a recently abandoned *renvall* that was used by the Rattuka group of the Gällivare forest Sami. Aronsson (1991) collected an oral history of the use of the site from Gunnar Nordvall, who gathered reindeer there, and whose grandmother before him used it for reindeer milking. When intensive herding was still in operation, reindeer were gathered here on a daily basis for milking over several consecutive weeks during the summer. One family or group of herders would own up to 20 pens within their pasturing area. The pens were rotated, each being used for several consecutive years. Rotation avoids the spread of reindeer diseases such as foot and mouth disease (Östlund et al. 2003) or calf diphtheria that infest the muddy, trampled soils and which affect calving in the pens to this day. A severe case of a similarly transmitted reindeer disease led to the abandonment of the Akkajärvi *renvall* around 1910–1920. Nordvall indicated that the pen was re-used for calf marking from ~1960–1970, by which time extensive reindeer herding was being applied. This required an expansion of the *renvall*, including the addition of an annex and a new hut (phase 2 on Fig. 2). Some large *Picea* were left standing within the *renvall* to provide shade for the reindeer. The *renvall* was abandoned altogether once the wider area became disrupted by tree felling activities during the 1980s. The duration of use of the *renvall* prior to its abandonment around 1920 is unknown.

### Site description

The *renvall* is situated at 386 m a.s.l. on the perimeter of a small fen measuring ~175 × 120 m, adjacent to Akkajärvi. The site forms part of a plateau between the Kalix-älven and Råne-älven rivers, in a region with mountains which are typically >500 m a.s.l. Approximately half of the area consists of morainic hills and ridges, while the remainder is covered by lakes and fens. Extensive felling of the boreal forest has taken place in the region, but the study site itself has not been directly affected.

Forest vegetation communities in northern Sweden are generally classified according to their ground layer composition, which at Akkajärvi is of the *Vaccinium vitis*-*idaea* or *V. myrtillus*-type with abundant ground lichens. The surrounding old growth forest is dominated by *Picea* and *Betula* spp. Hummocks on the fen are occupied by *Sphagnum*, *Empetrum* and *Vaccinium* with scattered occurrences of *P. sylvestris* and *Betula* spp., and Cyperaceae (mainly *Eriophorum angustifolium*) in the hollows. Poaceae and *Vaccinium* dominate the dry land ground layer, along with a scattering of *Epilobium*, *Silene dioica*, *Gnaphalium sylvaticum* and *Solidago virgaurea*. *Betula* spp. and *Pinus* saplings are becoming established in the old phase 1 hut area (Fig. 2). This suggests that the abandoned *renvall* is in the early stages of a post-disturbance secondary succession (Freschet et al. 2014).

## Methods

### Sample collection

**Fig. 3:**
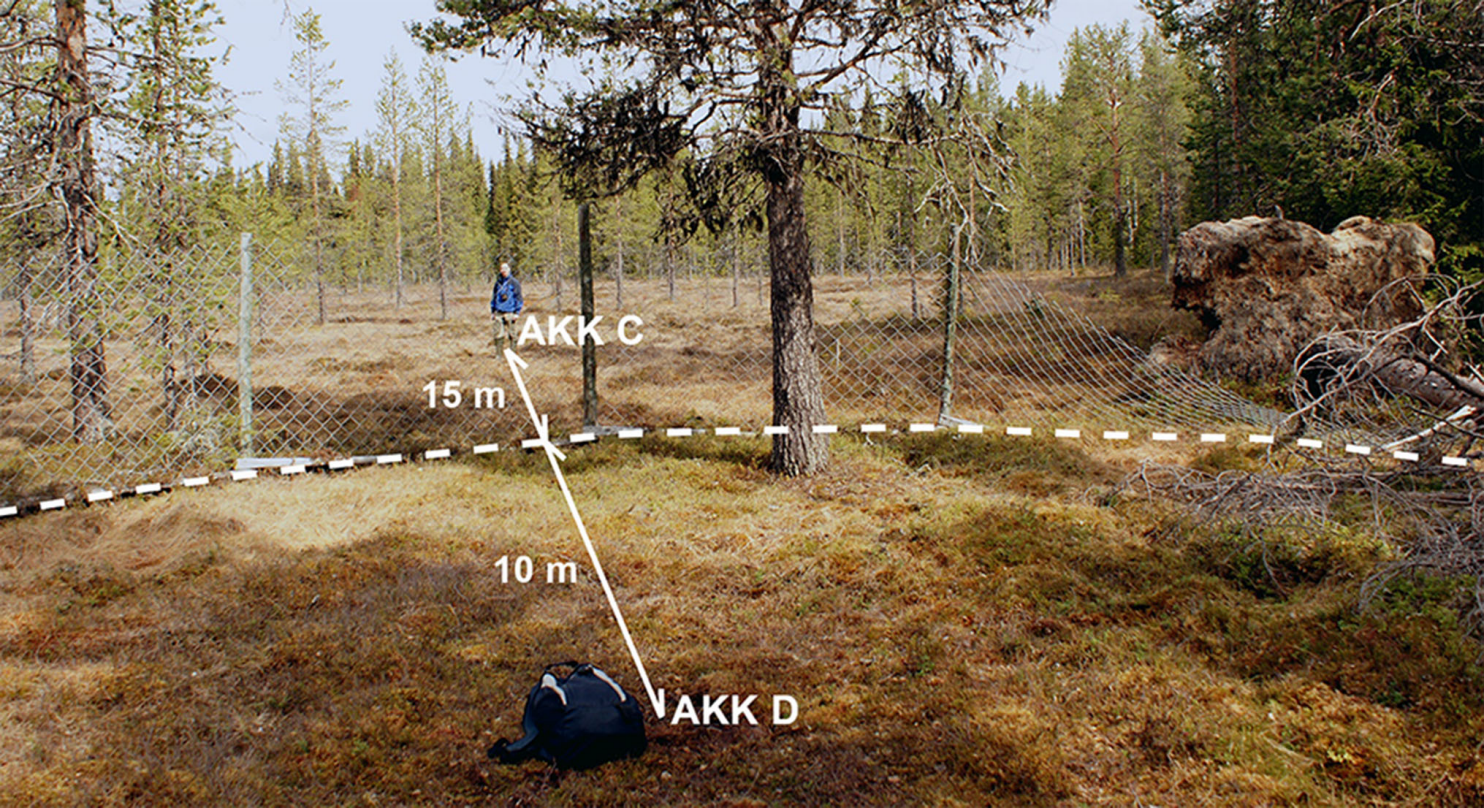
Photograph showing the relationship between the two sampling locations, Akkajärvi D (AKK D, inside the annexe) and Akkajärvi C (AKK C, out on the fen). The fence line/boundary to the annex of the *renvall* is indicated by a *dashed line*. The image is taken inside the annex facing north. (Photograph by I.M. Kamerling 2010)

Two peat profiles were collected to the west of the *renvall* (Fig. 2), at the base of a gentle slope. The first sequence, AKK D, was collected in a monolith tin from the open face of a soil pit dug within the annex to the *renvall* (66°52.896′N, 21°06.597′E), where reindeer were separated from the main herd during the extensive herding phase. It was hoped that the palynomorph record of this peat would contain a clear signal for reindeer herding, although there was some concern that stratigraphic integrity might have been affected through trampling by the animals. Therefore a second (paired) sequence, AKK C (66°52.906′N, 21°06.601′E), was collected using a Russian corer from the fen approximately 15 m outside the annex boundary and 25 m from AKK D (Figs. 2, 3). This profile, though less likely to be disturbed, was anticipated to contain a more muted record of cultural impact.

### Sedimentary characteristics

The stratigraphy of the peat sequences was described using the Troels-Smith (1955) scheme. In order to detect small changes in the minerogenic content of the highly organic peat, LOI was performed by Thermogravimetric Analysis (TGA), which measures weight loss through combustion in a controlled environment to determine the percentage of inorganic matter by weight (Ball 1964; Beaudoin 2003). Analyses were conducted using a Leco Corporation TGA-601 in the Sediment Analysis Laboratory at the Vrije Universiteit in Amsterdam. Contiguous 1 cm samples were dried in an oven at 80 °C overnight, ground to a powder, heated to 105 °C to expel H_2_O, weighed, and combusted at 550 °C until weight loss had ceased, usually ~3 h.

### Palynology

Contiguous 1 cm thick samples of ~1 cm^3^ were measured by volumetric displacement (Mooney and Tinner 2011). *Lycopodium* tablets were added to allow the determination of palynomorph concentrations (Stockmarr 1971). Pollen sample preparation followed conventional methods (Moore et al. 1991; Chambers et al. 2011). Samples were mounted unstained in silicone oil (12,500 cSt viscosity) and counted to a sum of ≥500 total land pollen (TLP) using a Nikon E400 binocular light microscope at 400× magnification and 600× for critical identifications. Obligate aquatic taxa, spores and exotic long-distance derived pollen types were excluded from the pollen sum. Pollen and spores were identified using the key in Moore et al. (1991) and the reference collection held in the Department of Geography and Environment, University of Aberdeen. Nomenclature largely follows Bennett (2015a). *Betula* pollen were measured, with those <20 μm classified as *B. nana* (dwarf birch), and grains above this size threshold regarded as tree birch (Mäkelä and Hyvärinen 1998). The uncertainties of this procedure are appreciated fully (Caseldine 2001), but given the low numbers of pollen grains assigned to *B. nana*, this is not considered to be critical.

Coprophilous fungal spores were identified using the notes and photographs in van Geel et al. (2003) and are prefixed HdV- (Hugo-de-Vries laboratory, University of Amsterdam; Feeser and O’Connell 2010). Coprophilous fungal spores are expressed as a percentage of the TLP sum. Microscopic charcoal was quantified through area measurement (Patterson et al. 1987; Conedera et al. 2009; Mooney and Tinner 2011). Only black, opaque, angular particles with a length ≥5 μm were considered (Patterson et al. 1987; Clark 1988). Charcoal concentrations (cm^2^ cm^−3^) were calculated to enable charcoal to pollen ratios (C:P; cm^2^ grain^−1^) to be determined. Calculations of rarefaction—a measure of estimated taxon richness—were made using psimpoll (Bennett 2015b).

Pollen data were collated using Tilia 2.0.b.4 software, and diagrams showing selected taxa were created using TGView 2.0.2 (Grimm 1990). Rare taxa that occurred at trace values (<1%) are indicated by a + symbol in the percentage diagrams. The placement of local pollen assemblage zones (LPAZs) was assisted through cluster analysis of the terrestrial pollen taxa using CONISS (Grimm 1987, 1990). Diagrams showing pollen accumulation rates (PAR or influx, measured as palynomorphs cm^−2^ year^−1^) were employed to provide absolute and independent measures of pollen abundance for selected taxa (Davis and Deevey 1964; Seppä and Hicks 2006). Taxa omitted from the selected palynomorph diagrams are shown in ESM Figs. 1, 2.

### AMS ^14^C dating

Peat samples were disaggregated overnight in 10% NaOH, sieved through a nest of 250, 180 and 120 µm meshes and residues were inspected using a Nikon SMZ645 stereoscopic zoom microscope at 8–50×. Selected plant macrofossils were removed from the sample residues using fine forceps and stored in plastic vials containing distilled H_2_O and a drop of 10% HCl. Where suitable terrestrial macrofossils were unavailable, the humic acid fraction of bulk (~1 cm^3^) peat samples was used. Samples were dated at the Scottish Universities Environmental Research Centre (SUERC), East Kilbride. All radiocarbon dates featured in this paper are calibrated using CALIB Version 7.0html (Stuiver and Reimer 1993; Stuiver et al. 2005) and the IntCal13 calibration curve (Reimer et al. 2013), and are reported at the 2σ confidence level.

### ^210^Pb dating

Contiguous samples of a known volume were dated at 1 cm resolution. Pre-treatment involved determining the wet bulk density, drying in an oven overnight at 40 °C, and calculating the dry bulk density (Foster et al. 2007). Samples were ground to a powder in a ball grinder and packed into cleaned and pre-weighed 7 × 1 cm OD PTFE cylinders, which were filled to 4 cm height (if <4 cm, the height of the sediment was recorded). The cylinders were sealed with paraffin wax and stored for at least three weeks before analysis to allow unsupported ^210^Pb to equilibrate with ^222^Rn (Appleby et al. 1986). ^210^Pb samples were measured at the University of Northampton and errors were determined according to the constant rate of supply (CRS) model.

### Age-depth models

In order to construct appropriate chronologies for the profiles under investigation, age-depth models were produced for comparison using both ‘classical’ age-depth modelling software (Clam; Blaauw 2010) and Bayesian techniques (Bacon; Blaauw and Christen 2011). Within Clam, various model settings were explored (each run with 10,000 iterations) and those with the best ‘goodness of fit’ were selected. Radiocarbon dates that appeared anomalous, for example those causing age reversals, were omitted. In Bacon, models were run (>6.5 million iterations) using different combinations of prior settings for deposition rate and accumulation shape. Priors were adjusted so that the model intersected the bulk of the probability distributions of the calibrated radiocarbon age ranges. The deposition rates used fall within the range that is considered reasonable for mires, based on recommendations in Mauquoy et al. (2002) and Goring et al. (2012). The precise details of model settings are provided in the relevant results sections. Calendar age ranges drawn from the models are reported in the text at the 2σ confidence level.

### Ordination

Multivariate statistics were limited to indirect gradient analysis through unconstrained ordination (DCA, PCA), executed using CANOCO 4.5, which combines ordination and multiple regression to solve questions about community ecology. DCA applied to untransformed percentage data for both cores revealed principal axes with lengths <2 s.d., and therefore PCA was selected as a more appropriate method of ordination (Ter Braak 1995; Ter Braak and Smilauer 2002). Analyses included taxa that consistently occurred at >1% of TLP. Indicator taxa for reindeer herding, as listed by Aronsson (1991), were also included, even if these pollen types only occurred at trace values. The data were log-transformed to improve the legibility of the ordination plots.

## Results

### Akkajärvi D (AKK D), inside the *renvall*

#### Lithostratigraphy

The lithostratigraphy at AKK D is described in Table 1. The clay-rich basal sand is probably of glacial origin and is overlain by an uninterrupted sequence of bryophyte-rich peat.

**Table 1:** Lithostratigraphy of the Akkajärvi D and C (AKK D, AKK C) sequences described using Troels-Smith (1955) formulae and written descriptions. Only the sections above 27 cm (AKK D) and from 23–0 cm (AKK C) were pollen-analysed

Depth (cm)	Troels-Smith formula	Unit description
AKK D		
23–0	TSphag^3^3 Tl^1^1 Th^1^+ Sh+ Nigr 3 Strat 0 Sicc 3 Elas 1 Lim n/a	Variously-humified yellow–brown bryophyte peat, containing abundant woody rootlets and traces of herbaceous rootlets and sand
28–23	Sh3 As1 Th+ Dl+ Anth+ Nigr 3+ Strat 0 Sicc 3 Elas 0 Lim 0	Well-humified dark-brown clay-rich peat, containing traces of herbaceous rootlets. 28–26 cm black in colour
31–28	Gmin3 As1 Nigr 2 Strat 0 Sicc 3 Elas 0 Lim 0	Light-grey clay-rich sand
AKK C		
20–0	TSphag^1^3 Th^1^1 Tl^1^+ Dl+ Nigr 1+ Strat 0 Sicc 3 Elas+ Lim n/a	Poorly-humified yellow to mustard-brown *Sphagnum* peat, containing traces of herbaceous and woody rootlets, and woody detritus
38–20	As2 Th^3^2 Sh+ Tl^3^+ Nigr 3+ Strat 0 Sicc 3+ Elas 0 Lim 1	Well-humified dark brown/black peat, containing increasing clay content down the profile. Herbaceous stems and rootlets, plus traces of woody rootlets

#### Chronology

Radiocarbon and ^210^Pb dates for AKK D are presented in Tables 2 and 3. SUERC-27810 (28–27 cm) appears erroneous, as it is too old compared to the age of the bryophytes above it, and was omitted from the age-depth model. It is possible that this piece of charcoal, coming from the interface between the sandy basal sediment and the overlying peat, was deposited on the old land surface before the onset of paludification. The uppermost three ^14^C dates returned indistinguishable calibrated age ranges (approximately cal 1650–1950) due to reversals in the IntCal13 calibration curve (Reimer et al. 2013). Bayesian age-depth modelling with Bacon operates with the prior assumption that underlying samples must be older than those above them. The inclusion of SUERC-23898 and -23899 within Bacon enables the chronology to be extended beyond the ^210^Pb series.

**Table 2:** Radiocarbon dates and ±2σ calibrated age ranges for the Akkajärvi D and C (AKK D, AKK C) sequences, calibrated with CALIB 7.0 (Stuiver et al. 2005), operating IntCal13 (Reimer et al. 2013)

Depth (cm)	Lab code	Material	^14^C yr BP (±1σ)	Cal. age (±2σ)	δ^13^C (‰)
AKK D					
13–12	SUERC-23897	Bryophyte stems and leaves	135 ± 30	ad 1672–1942	−27.7
18–17	SUERC-23898	Bryophyte leaves	65 ± 30	ad 1693–1955	−21.5
23–22	SUERC-23899	Bryophyte leaves	85 ± 30	ad 1687–1926	−23.9
28–27	SUERC-27810	Charcoal	2,360 ± 30	534–383 bc	−26.4
AKK C					
20–19	SUERC-27808	Bryophyte leaves	105 ± 30	ad 1681–1954	−23.7
25–24	SUERC-23894	Peat (humic acid)	140 ± 30	ad 1669–1953	−27.6
29–28	SUERC 27809	Peat (humic acid)	605 ± 30	ad 1297–1405	−28.0
34–33	SUERC-23895	Peat (humic acid)	1,175 ± 30	ad 774–964	−27.8
37–36	SUERC-23896	*Betula* twig	1,705 ± 30	ad 225–408	−30.4

**Table 3:** ^210^Pb CRS (constant rate of supply) ages for the Akkajärvi D and C (AKK D, AKK C) sequences and their ±1σ error

Depth (cm)	CRS age (cal ad)	CRS age error (± 1σ)
AKK D		
0	2008	0
1–0	2004	1
2–1	2000	2
3–2	1992	2
4–3	1987	2
5–4	1984	2
6–5	1980	2
7–6	1976	3
8–7	1968	3
9–8	1958	4
10–9	1951	4
11–10	1943	5
12–11	1932	6
13–12	1924	8
14–13	1915	10
15–14	1890	20
AKK C		
0	2008	0.00
1–0	2008	1.00
2–1	2005	1.09
3–2	2003	1.25
4–3	2001	1.31
5–4	1999	1.44
6–5	1997	1.49
7–6	1995	1.57
8–7	1991	1.64
9–8	1988	1.72
10–9	1986	1.77
11–10	1983	1.83
12.5–11	1982	1.88
13.5–12.5	1978	2.00
15–13.5	1972	2.17
16–15	1962	2.46
17–16	1958	2.59
18–17	1955	2.61
19–18	1952	2.75
20–19	1946	2.91
21–20	1938	2.77
22–21	1923	6.42
23–22	1910	3.41
24–23	1891	5.79
25–24	1864	19.48

**Fig. 4:**
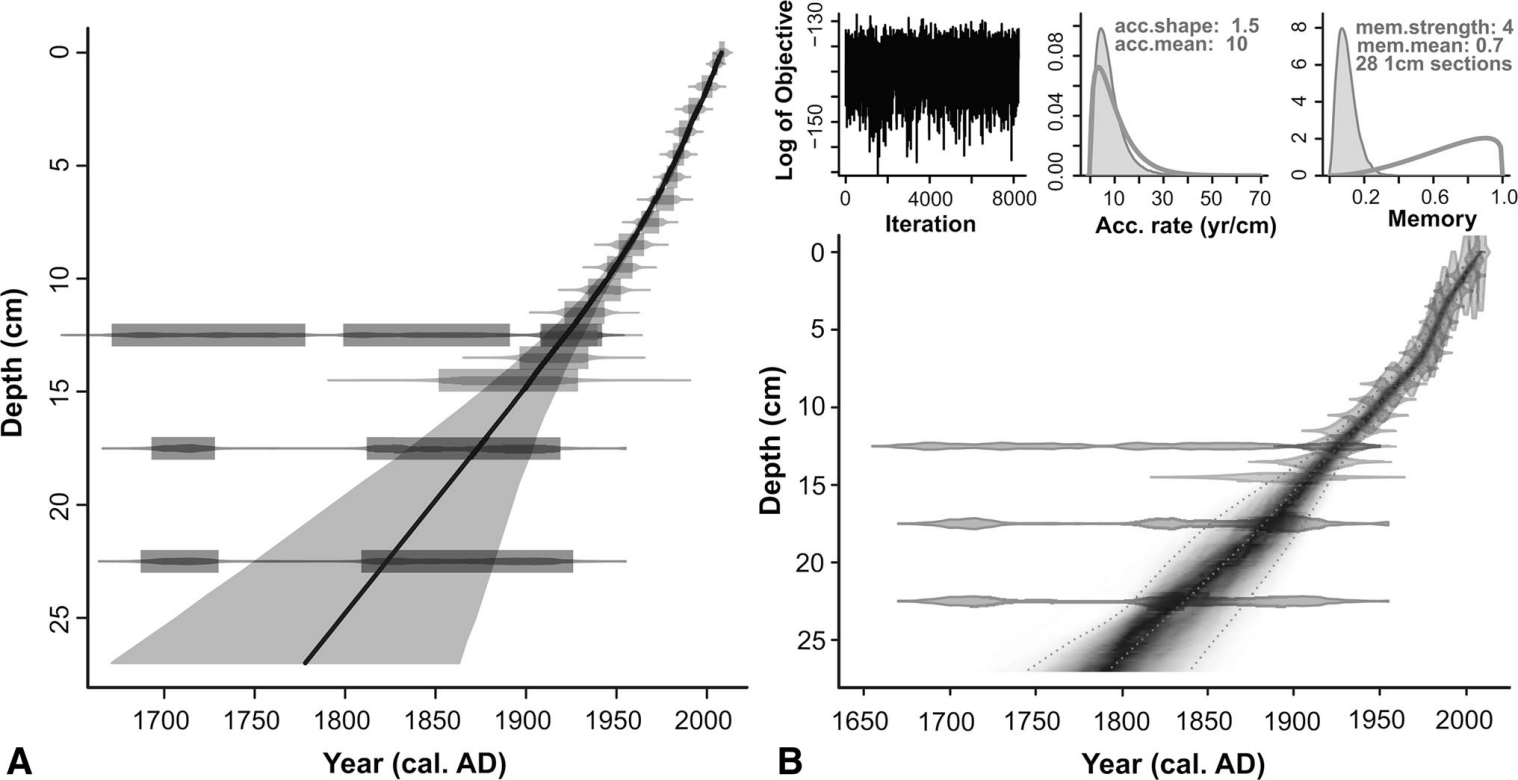
Age-depth models for Akkajärvi D produced using **a** Clam (Blaauw 2010) and **b** Bacon (Blaauw and Christen 2011). Both models consider all radiocarbon measurements on bryophytes plus the ^210^Pb dates (Table 2, 3). The basal ^14^C date on charcoal (SUERC-27810; 28–27 cm) was omitted because it was considered to be erroneously old (see text for further explanation). The best ‘goodness of fit’ in Clam (12.15) was achieved by fitting a smoothed spline. In Bacon the following priors were set: deposition rate (acc.mean) of 10 year cm^−1^; accumulation shape (acc.shape) of 1.5; memory strength (mem.strength) of 4; memory mean (mem.mean) of 0.7. The model extrapolates to the base of the pollen-analysed sequence (27 cm)

The age-depth models produced with Clam and Bacon (Fig. 4a, b) are near identical. Preference was given to the model produced with Bacon, where the age envelope for the period of greatest interest here, the last few centuries, displays more conservative estimates. The model suggests that the top 28 cm of peat covers the past ~200 calendar years. This is in broad agreement with findings obtained at a variety of sites in inland northern Norrland, where the top 20–30 cm of profiles generally consist of poorly-humified peat not older than 200–300 years (Bradshaw and Zackrisson 1990; Aronsson 1991).

#### Palynology

**Fig. 5:**
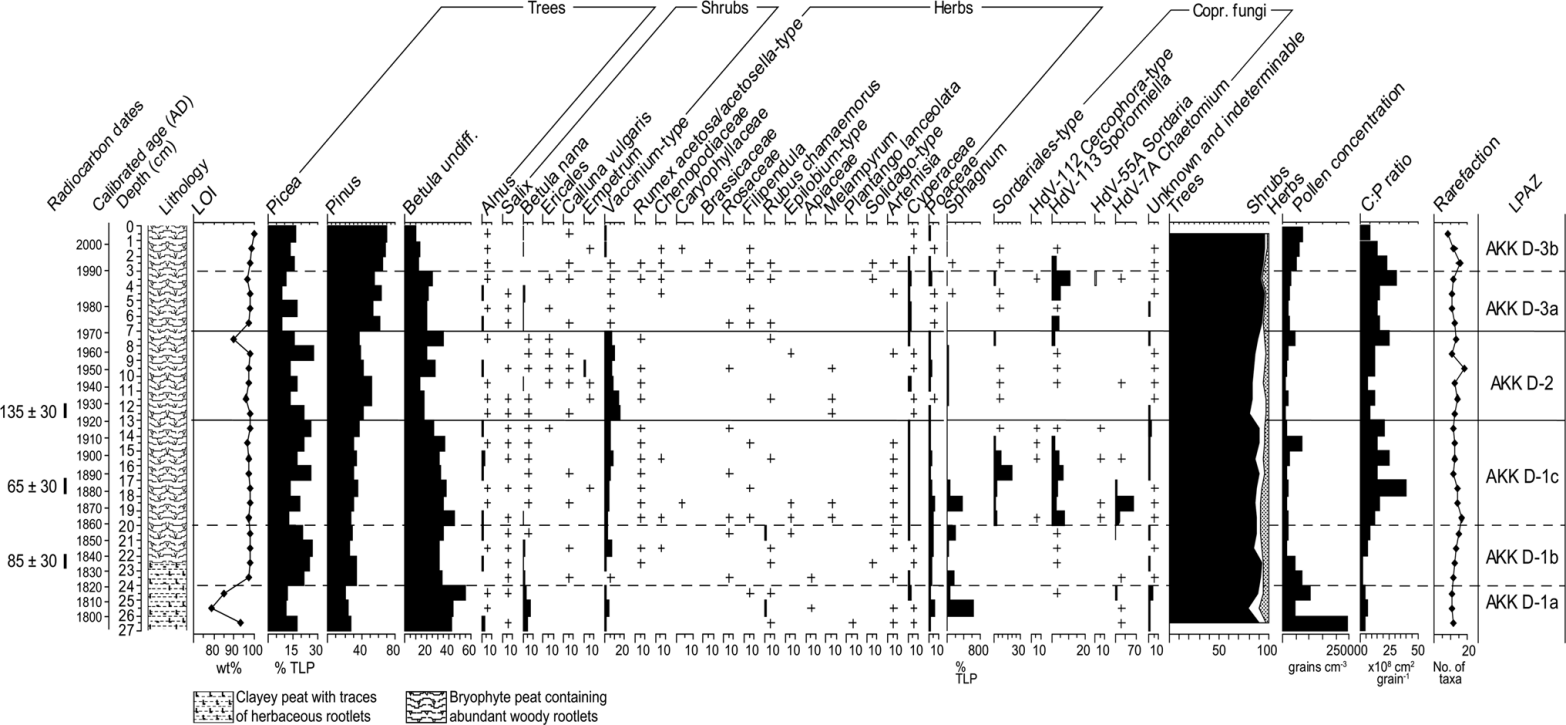
Percentage pollen diagram for Akkajärvi D (AKK D) showing selected trees, shrubs, herbs, *Sphagnum* and coprophilous fungi. X-axis units are % TLP (total land pollen; minimum sum of 500) unless stated otherwise. Also included are the uncalibrated ^14^C dates, a calibrated timescale (ad) based on the age-depth model (Fig. 4, panel B), the lithological column for the sequence, the weight percentage (wt%) loss-on-ignition (LOI) values, the summary diagram, microscopic charcoal expressed as charcoal to pollen (C:P) ratio in cm^2^ grain^−1^ and the rarefaction index (number of taxa), with a maximum variance of ± 2 taxa; rare types (<1%) are indicated by a + symbol

**Fig. 6:**
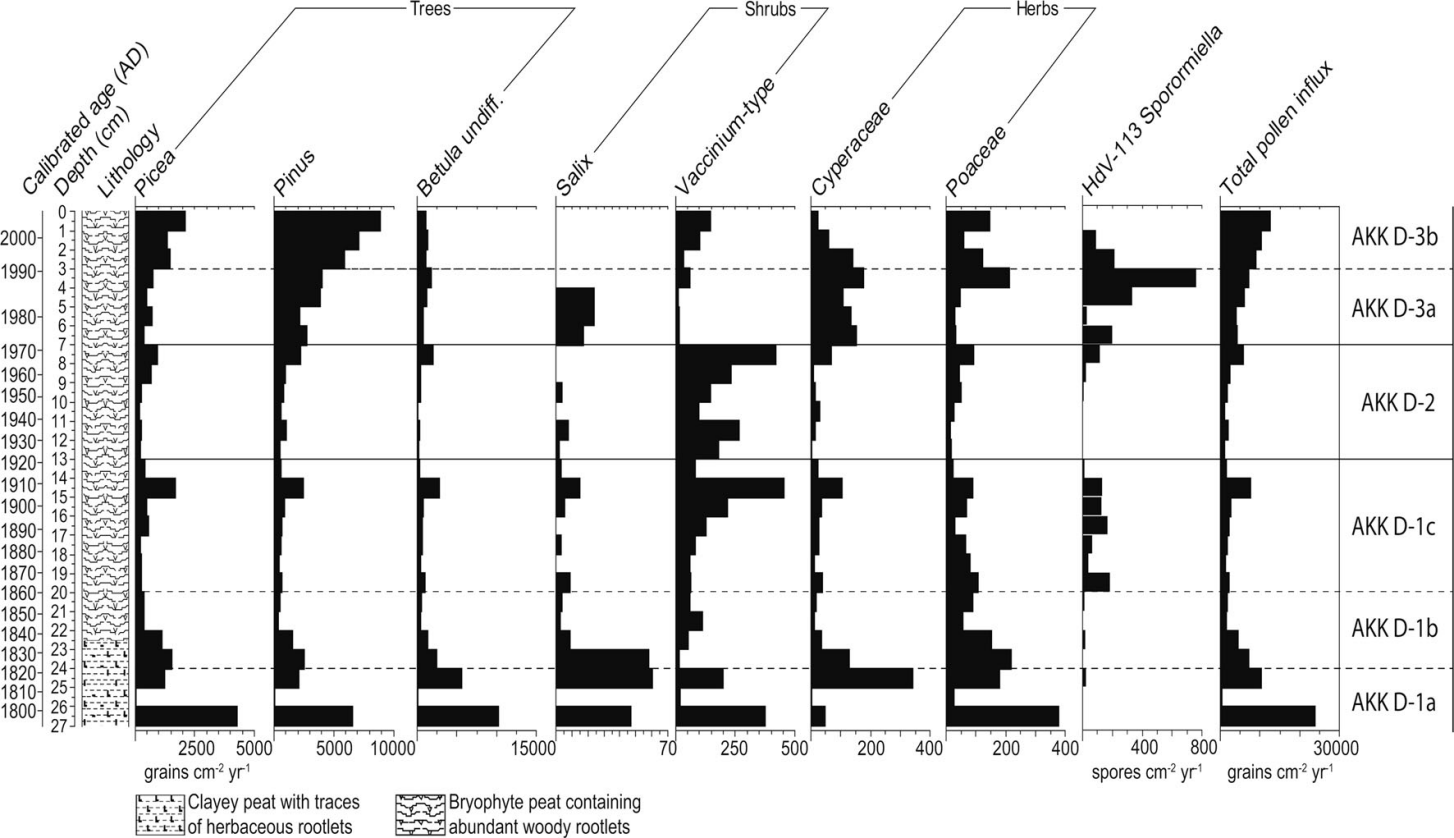
Pollen accumulation rates measured in grains cm^−2^ year^−1^ for selected trees, shrubs and herbs and spore accumulation rates (spores cm^−2^ yr^−1^) for *Sporormiella*-type at Akkajärvi D (AKK D). Note the differences in scaling of the x-axes. Also included are the uncalibrated ^14^C dates, a calibrated timescale (ad) based on the age-depth model (Fig. 4, panel B) and the lithological column for the sequence

Trampling in the annex during the extensive reindeer herding phase does not appear to have caused deleterious homogenization of the profile and its contained artefacts; the pollen spectra (Figs. 5, 6) seem to retain their stratigraphic integrity and display reasonably sharp and intelligible changes. Three LPAZs can be distinguished (Figs. 5, 6), and their key features are summarised in Table 4.

**Table 4:** Description of the LPAZs at Akkajärvi D and C based on percentage and PAR diagrams, including loss-on-ignition (LOI), the charcoal to pollen ratio (C:P) and pollen concentrations

LPAZ (AKK)	Depth (cm)	Cal age (ad)	Diagnostic pollen and spore characteristics based on percentage and PAR diagrams
D-3b	3–0	1990–2009	*Pinus* and *Picea* increase (both in relative and absolute terms); *Vaccinium*-type recovers; Cyperaceae are reduced, Poaceae retain near-constant values, no other herbaceous taxa present at 1–0 cm; C:P falls throughout, rarefaction values decrease from 15–8 taxa and LOI increases to 99%; pollen influx and concentrations further increase
D-3a	7–3	1970–1990	*Pinus* gains dominance (38–52%); *Vaccinium*-type reduced to traces; Cyperaceae increase by ~200 grains cm^−2^ yr^−1^, while Poaceae are mostly reduced to trace values and traces of Chenopodiaceae and *Solidago*-type re-emerge at 5–3 cm; *Sporormiella*-type (HdV-113); C:P increases and rarefaction values fluctuate between 11 and 13 taxa; pollen concentrations and influx increase somewhat
D-2	13–7	1920–1970	PAR of all tree taxa increase; *Vaccinium*-type increases and pollen of other ericaceous shrubs (i.e. *Calluna*, *Empetrum*) becomes more common; Cyperaceae are regularly reduced to trace values; Cyperaceae mostly reduced to trace values. Fewer occurrences of herbaceous taxa, most notably *Artemisia*; frequencies of coprophilous fungal spores are reduced to trace values with intermittent presence; C:P reduced, rarefaction values fluctuate between 11 and 14 taxa, with an excursion to 18 taxa at 10–9 cm, and the LOI dips to 90% at 8–7 cm
D-1c	20–13	1860–1920	*Picea* and *Pinus* increase, which is balanced out by a decline in *Betula* undiff.; *B. nana* reduced to traces as *Vaccinium*-type increases; Poaceae (PAR) fluctuates and traces of a greater range of herbs (e.g. Caryophyllaceae and *Melampyrum*-type) first appear, whilst Apiaceae and *Solidago*-type disappear; coprophilous fungal spores of Sordariales, *Sporormiella*-type (HdV-113) and *Chaetomium*-type (HdV-7a) become abundant; rarefaction increases to 17 but then decreases to 12 taxa and LOI remains high and stable (~98%)
D-1b	24–20	1825–1860	All trees decline (PAR) while *Vaccinium*-type increases; Cyperaceae and Poaceae decrease (PAR), traces of *R. acetosa*/*acetosella*-type, Chenopodiaceae, *Epilobium*-type and *Solidago*-type appear for the first time; traces of *Sporormiella*-type (HdV-113) present; C:P increases somewhat, rarefaction values increase from 12 to 15 taxa, LOI high and stable (~98%); pollen influx and concentrations decline
D-1a	Up to 24	Up to 1825	*Betula* undiff. dominant (>40% TLP) with *Picea* and *Pinus* contributing a further ~40%; prominent non-arboreal taxa include *B. nana*, *Vaccinium*-type, Cyperaceae and Poaceae; rarefaction consequently low (10–12 taxa), as are C:P and LOI shows a mid-zone decline to 79%
C-4	4–0	2000–2009	*Pinus* rises to dominate (>60%); *B. nana* reduced; Many herbaceous taxa disappear and traces of *R. acetosa/acetosella*-type, Chenopodiaceae, *Spergula*, Rosaceae and *Rubus chamaemorus* are discontinuous; coprophilous fungal spores become infrequent; C:P reduced and rarefaction values decrease (9–14 taxa)
C-3b	7–4	1995–2000	All trees are reduced while the influx and percentages of *B. nana* and *Vaccinium*-type become slightly elevated; Herbaceous flora diversify (e.g. *Epilobium*-type and *Plantago major* are added), Cyperaceae are reduced traces and Poaceae increases; coprophilous fungal diversify and increase; C:P increases and rarefaction peaks mid-zone at 18 taxa
C-3a	14-7	1975–1995	*Pinus* and *Picea* increase; Fewer herbaceous taxa occur, and frequencies of Poaceae and Cyperaceae are significantly reduced, while *Ranunculus acris*-type and *Galium*-type appear; coprophilous fungal spores become rare, while *Sphagnum* peaks. C:P is reduced, rarefaction values decrease from 17 to 12
C-2	20–14	1945–1975	Reductions in *Pinus* and *Picea*, *Betula* now dominates; *Vaccinium*-type peaks then falls; Poaceae increase and many herbs appear at trace values (*R. acetosa/acetosella*-type, Chenopodiaceae, Brassicaceae, *Epilobium*-type, *Melampyrum*, *Plantago lanceolata*, *Achillea*-type, *Artemisia*). *Sporormiella*-type (HdV-113) elevated, traces of Sordariales, *Sordaria* (HdV-55A) and *Chaetomium* (HdV-7A). C:P elevated, rarefaction increases (13–20 taxa) and LOI decreases to 95% at 15–14 cm
C-1	Up to 20	Up to 1945	*Pinus*, *Picea* and *Betula* undiff. dominant; *B. nana* and *Vaccinium*-type low; limited herbaceous pollen (notably *R. chamaemorus*, Cyperaceae and Poaceae with traces of *Solidago*-type and *Artemisia* at 21–20 cm). Traces of Sordariales, *Sporormiella*-type [HdV-113] and *Chaetomium* [HdV-7A]; C:P low, rarefaction at 12–13 taxa

### Akkajärvi C (AKK C), outside the *renvall*

#### Lithostratigraphy

The profile at AKK C consists of an uninterrupted peat deposit (Table 1). In the field, this was proven with a gouge auger to rest on a base of coarse sand at ~60 cm depth. The lower peat unit within the monolith (below 20 cm) is well-humified and contains trace amounts of clay. Poorly-humified bryophytes form the dominant component in the topmost 20 cm. This is broadly comparable to the lithostratigraphy recorded at AKK D (Table 1).

#### Chronology

**Fig. 7:**
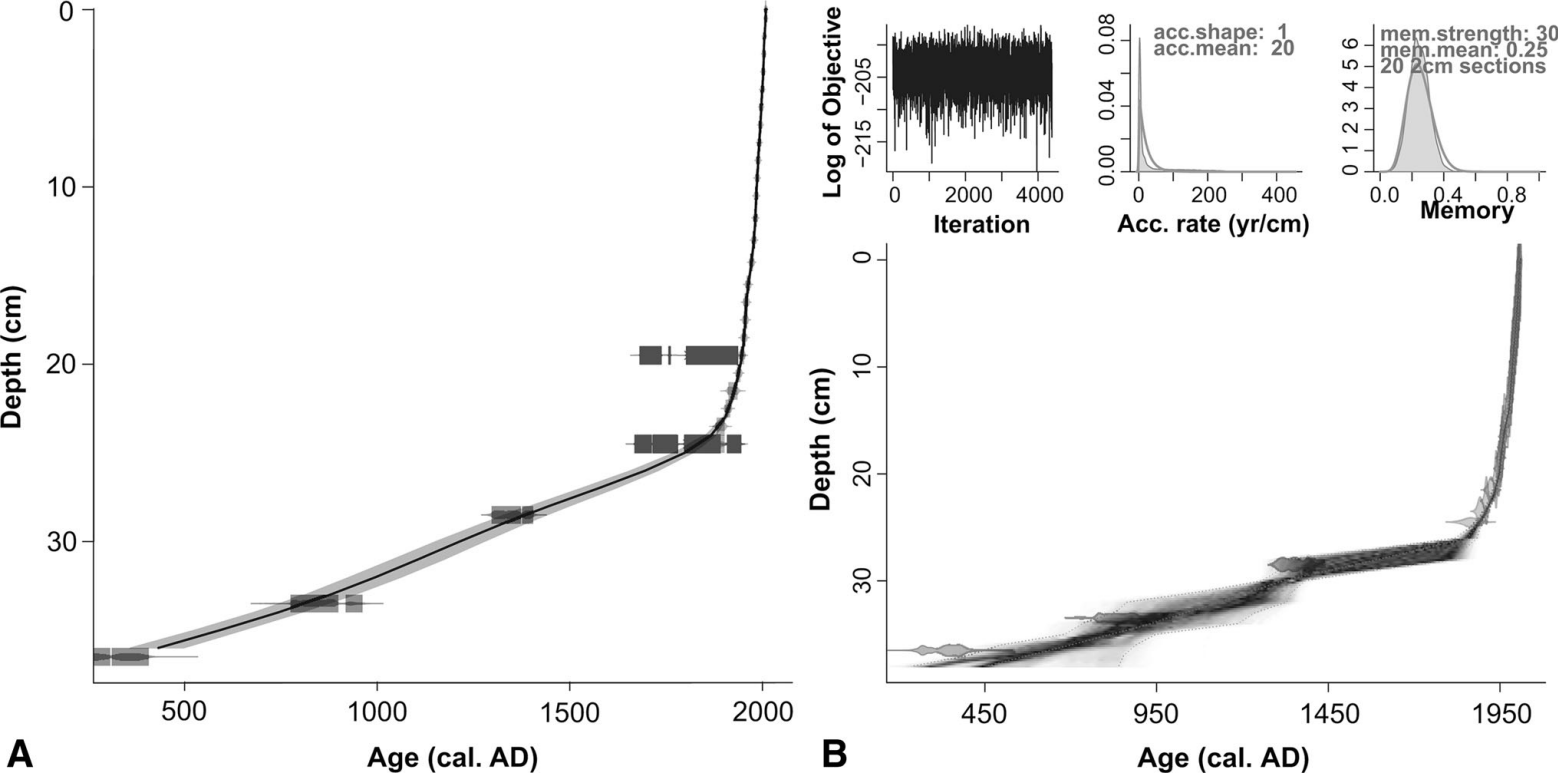
Age-depth models for Akkajärvi C produced using **a** Clam (Blaauw 2010); **b** Bacon (Blaauw and Christen 2011). Both models consider ^14^C measurements on peat samples (humic acid fraction) at 29–28 and 34–33 cm (SUERC-27809 and 23895 respectively), a *Betula* twig at 37–36 cm (SUERC-23896), plus the ^210^Pb dates (Tables 2, 3). Radiocarbon dates on material at 25–24 cm (humic acid fraction of peat; SUERC-23894) and at 20–19 cm (bryophyte leaves; SUERC-27808) are marked as outliers because more accurate ^210^Pb dates were also available for these levels. The best goodness of fit in Clam (18.32) was achieved by fitting a smoothed spline, whereas in Bacon a prior deposition rate (acc.mean) set to 20 yr cm^−1^, an accumulation shape (acc.shape) of 1, a memory strength (mem.strength) of 10 and a memory mean (mem.mean) of 0.25, provided the best fit. A separate model was produced with Bacon for the pollen analysed section only (Fig. 8)

**Fig. 8:**
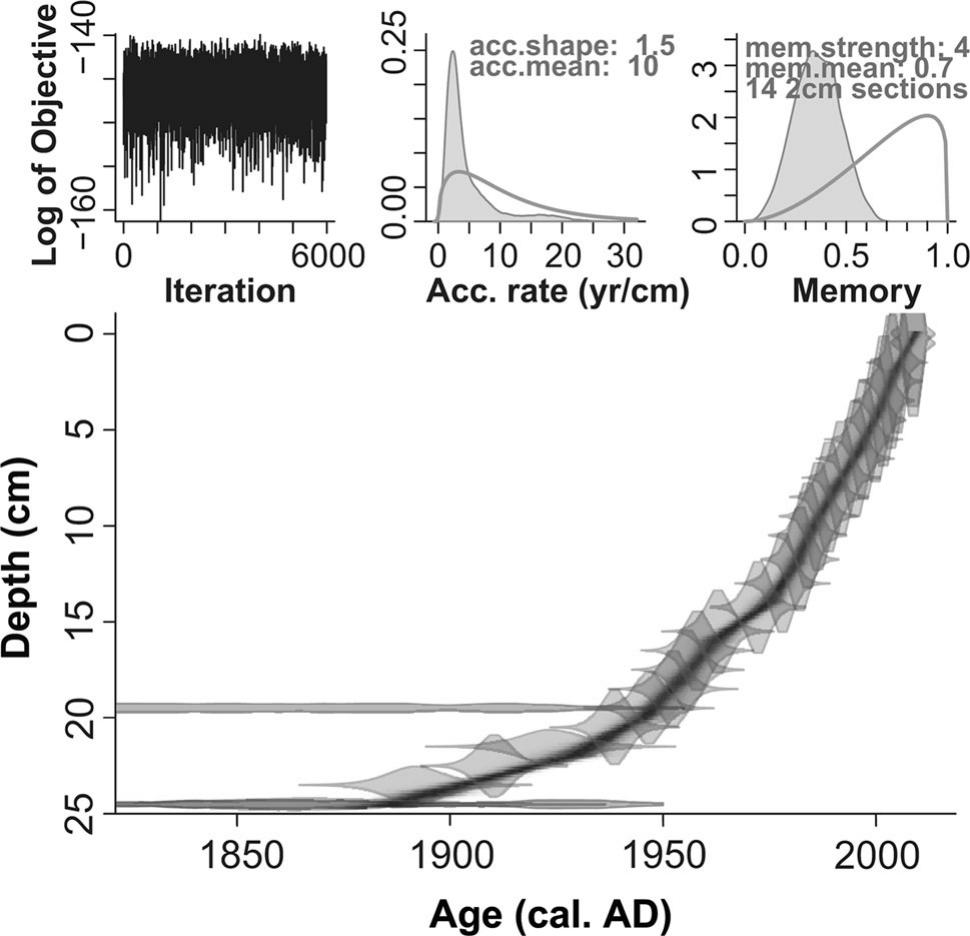
Bacon age-depth model covering the pollen-analysed sequence of Akkajärvi C (AKK C; 0–23 cm). Model priors were set as follows: deposition rate (acc.mean) = 10 yr cm^−1^; accumulation shape (acc.shape) = 1.5; memory strength (mem.strength) = 4; memory mean (mem.mean) = 0.7

Radiocarbon and ^210^Pb dates for AKK C are presented in Tables 2 and 3. Age-depth models produced with Clam and Bacon over the full radiocarbon-dated section of the profile (0–37 cm; Fig. 7) produce near-identical results and the latter (more conservative) model was selected. Both models show low accumulation rates below ~25 cm due to compaction and humification of the peat. This is in broad agreement with patterns for other mires throughout northern Sweden (Bradshaw and Zackrisson 1990; Aronsson 1991). The Bacon age-depth model was produced covering only the pollen-analysed section (0–23 cm; Fig. 8). This incorporates radiocarbon dates on the humic acid fraction of a peat sample and bryophyte leaves (SUERC-23894 [25–24 cm] and -27808 [20–19 cm] respectively) together with ^210^Pb dates. The latter help overcome the difficulty of relying solely on ^14^C dates which, when calibrated, span the problematic ad 1650–1950 section on the IntCal13 calibration curve noted earlier (Reimer et al. 2013).

#### Palynology

**Fig. 9:**
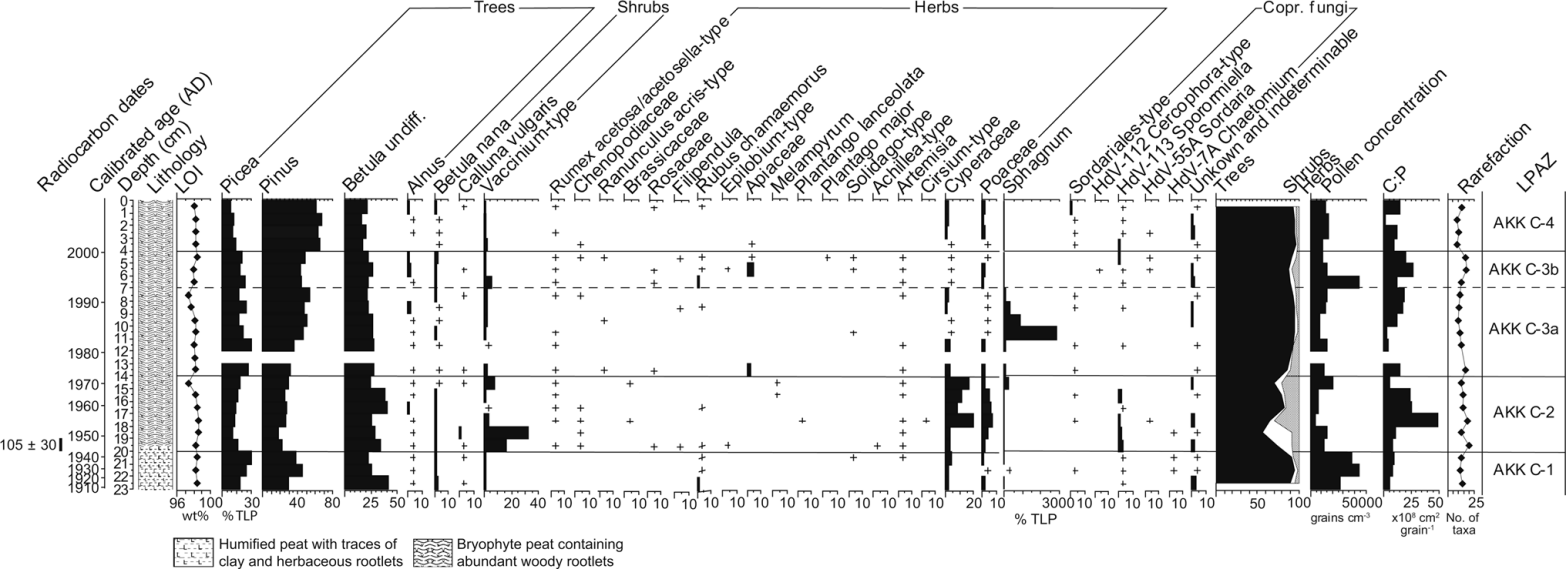
Percentage pollen diagram for Akkajärvi C (AKK C) showing selected trees, shrubs, herbs, *Sphagnum* and coprophilous fungi. Calculations are based on a minimum total land pollen (TLP) sum of 500. Also included are the uncalibrated ^14^C dates, calibrated ages (ad) based on the age-depth model (Fig. 8), the lithological column for the sequence, the weight percentage (wt%) loss on ignition (LOI) values, the summary diagram, microscopic charcoal expressed as charcoal to pollen (C:P) ratio in cm^2^ grain^−1^ and the rarefaction index (number of taxa), with a maximum variance of ±1 taxa; rare types (<1%) are indicated by a + symbol. Data are unavailable for 13–12 cm due to the low pollen concentration in this sample

**Fig. 10:**
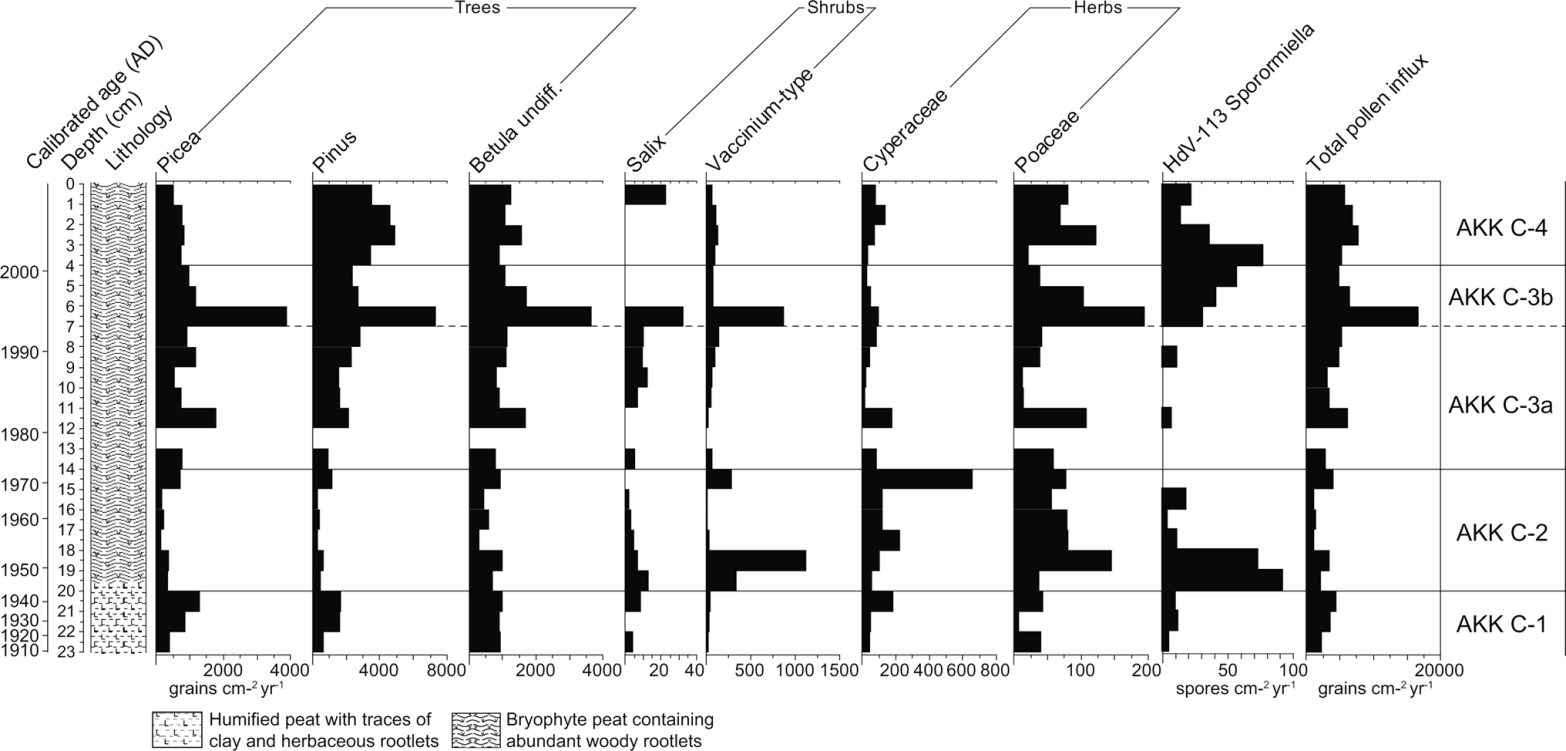
Pollen accumulation rates measured in grains cm^−2^ year^−1^ for selected trees, shrubs and herbs, and spore accumulation rates (spores cm^−2^ yr^−1^) for *Sporormiella*-type, at Akkajärvi C (AKK C). Note the differences in scaling of the x-axes. Also included are the uncalibrated ^14^C dates, a calibrated timescale (ad) based on the age-depth model (Fig. 8) and the lithological column for the sequence. Data for 13–12 cm are unavailable due to the low pollen concentration in this sample

Four LPAZs can be distinguished, with AKK C-3 further divided into two subzones (Figs. 9, 10). The key features of the pollen diagram are summarized in Table 4. The pollen-analysed section at AKK C only covers the top 23 cm (from ~1910 onward) of a 60 cm long core. Palynological analysis below 23 cm was not undertaken as it was considered unlikely to provide the desired information on the short-lived decadal-scale occupation activity at the site, due to low temporal resolution below ~25 cm as a result of slow peat accumulation rates and/or sediment compaction (Fig. 7).

### Ordination

**Fig. 11:**
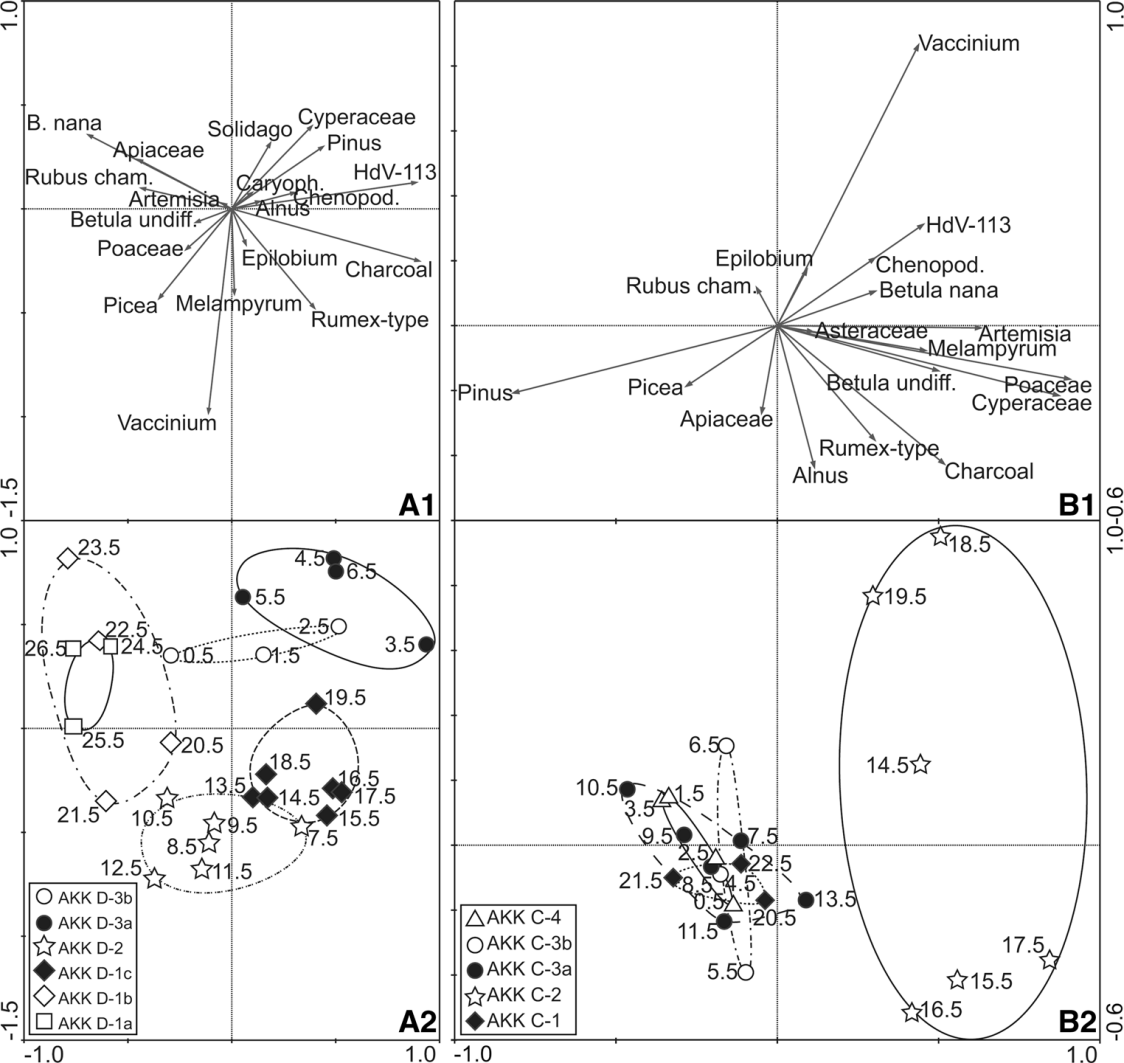
PCA scatterplots for Akkajärvi D (panels A1, A2) and Akkajärvi C (panels B1, B2). A1 and B1 depict palynomorphs and LOI. A2 and B2 display sample scores for the pollen assemblages and are grouped according to LPAZs

The first two PCA axes of AKK D explain 61.8% of the variance in the dataset of which 36.9% is explained by axis 1 (Fig. 11). The corresponding data for AKK C explain 58.8% of the variance with 38.0% explained by axis 1 (Fig. 11).

## Discussion

### Ordination

The results of the PCA analyses reveal structure within the palaeo-vegetation that is significant when considering the palynological data. For this reason, these data are discussed at the outset.

The 36.9% of the PCA variance explained by axis 1 for AKK D may reflect grazing intensity. The vector for HdV-113 (*Sporormiella*-type) extends to the positive end of this axis and correlates with several other indicators of reindeer herding, such as charcoal, Chenopodiaceae, Asteraceae, *Rumex*-type and *Epilobium*-type. Pollen samples from LPAZs AKK D-1c and -3a cluster in this section of ordination space. Taxa that correlate with the negative end of axis 1 include *B. nana*, *Rubus chamaemorus* and Apiaceae; these are considered by some to be grazing-sensitive (Scott 2001; Lendvay and Kalapos 2014; Landi et al. 2016) and may reflect periods when reindeer herding was inactive. A caveat is required, however, because Poaceae also records a negative score on axis 1. Samples from LPAZs AKK D-1a and -1b are associated with this area of the ordination space and therefore may reflect an absence of grazing, or at least fewer animals on site. Samples from LPAZs AKK D-2 and -3b plot towards the centre of axis 1, and may represent vegetation communities undergoing succession following the removal of grazing pressure.

The environmental control underlying axis 2 for AKK D, which explains 24.9% of the variance, is more difficult to explain, but it may represent a hydrological gradient. Certain taxa typically found on fen surfaces such as Cyperaceae plot towards the positive end of the axis, whilst taxa more representative of the drier soils inside the reindeer pen such as *Picea* and *Vaccinium* display negative vectors.

The 38% of the PCA variance explained by axis 1 for AKK C appears to reflect a landscape openness gradient. Vectors for *Pinus* and *Picea* point towards the negative end of the axis and seemingly represent closed canopy forest. Open ground indicators such as Poaceae, Cyperaceae and *Artemisia* plot towards the positive end of axis 1. *Betula* spp.—likely to be amongst the first trees and shrubs to invade abandoned areas—show positive axis 1 scores but have short vectors. The local abundance of *Betula* is currently very low and pollen accumulation rates at AKK D and C are generally <1,000 grains cm^−2^ yr^−1^, suggesting only sparse presence of tree birch at the site (Hicks and Hyvärinen 1999). Samples from LPAZs AKK C-1, -3a, -3b and -4 plot as a tight cluster on the negative (forested) side of axis 1, while samples from AKK C-2 sit at the positive (open landscape) end.

Axis 2 explains the remaining 20.8% of the variance and is difficult to interpret, but may reflect a hydrological gradient. The vector for *Alnus*, most likely *A*. *incana* based on the distribution patterns in Mossberg and Stenberg (2010), extends furthest along the negative side of axis 2. *A. incana* has a preference for poor, wet soils. Apiaceae displays a similar pattern. In northern Sweden this family includes taxa such as *Cicuta virosa* var. *virosa*, *Angelica sylvestris*, *A*. *archangelica* ssp. *archangelica* and *Peucedanum palustre* (Mossberg and Stenberg 2010), and these grow along watercourses and in damp environments. *Vaccinium* displays the longest vector aligned with the opposite (positive) end of axis 2. This could relate to *V. vitis*-*idaea* and *V. myrtillus*, both of which dominate the ground layer at the site today and are often associated with relatively dry situations; its habitats include, for example, coniferous forests, deciduous forests, heaths, pastures, protected hillsides, roadsides and crevices (Mossberg and Stenberg 2010). Alternatively, an increase in the availability of dry hummocks within the fen, where *Vaccinium* also tends to grow, could explain why it is positioned at the positive end of the axis.

### Vegetation and land use reconstruction

Given the similarities between AKK D and C, the palynological data from the former (within the *renvall*), with its superior time range, are interpreted here before drawing comparisons with AKK C. It was considered whether to draw analogies with previous studies of reindeer herding or (Mesolithic) hunter-gatherer impacts on the landscape, but such work in Fennoscandia has focused on different environmental settings, such as on high altitude alpine woodlands and treeless alpine heaths (Salmonsson 2003; Karlsson et al. 2007; Staland et al. 2011; Bergman et al. 2013; Möller et al. 2013) or on coastal areas (Hörnberg et al. 2005). Extensive logging from the end of the 1800s has destroyed archaeological evidence of both Sami reindeer herding and agricultural settlement (Östlund and Bergman 2006). Modern analogue studies of the impacts of reindeer trampling and grazing on the vegetation mainly consider the effects on lichen abundance in winter grazing areas on alpine tundras and in woodlands (Väre et al. 1995; Suominen and Olofsson 2000). The *renvall* at Akkajärvi, however, was not used for grazing but was strictly employed for gathering of reindeer in summer, first for milking and later for calf-marking.

#### Natural conditions in the absence of human impact (~1795–1825)

High arboreal pollen frequencies in AKK D-1a (>80% *Picea*, *Pinus* and *Betula* undiff.), a lack of palynological ‘indicators’ for reindeer herding (Aronsson 1991), the near-absence of coprophilous fungal spores and low charcoal to pollen (C:P) values combine to suggest that the *renvall* was not in use during this period. LOI values are very high compared to the succeeding LPAZs, implying that soils were stable. The elevated pollen influx values in the basal pollen sample seemingly reflect the dominance of high pollen producing trees such as *Pinus* and *Betula* (Hicks and Hyvärinen 1999).

#### Initiation of intensive clearance and gathering of animals (~1825–1860)

In AKK D-1b, low levels of HdV-113 (*Sporormiella*-type) spores and the appearance of traces of *Rumex*-type, Chenopodiaceae, *Epilobium*-type and *Solidago*-type pollen suggest that some level of disturbance occurred during this period. Pollen accumulation rates (PARs) for *Pinus* and *Betula* also drop below the threshold for local presence, <2,000 and <1,500 grains cm^−2^ yr^−1^ respectively in northern Fennoscandian boreal forests (Hicks and Hyvärinen 1999) and C:P values increase somewhat, although not far beyond the values recorded for AKK D-1a. The former may be related to some initial clearance within the *renvall* in preparation for reindeer herding, and the increase in C:P could be due to the input of microscopic charcoal produced by smudge and/or domestic fires.

#### Intensive reindeer herding (~1860–1920)

High frequencies of coprophilous fungal spores (*Sporormiella*-type, Sordariales-type and *Chaetomium*-type) in AKK D-1c, together with elevated C:P, provide strong evidence for a period of reindeer herding activity with smudge fires at the site. A slight increase in Cyperaceae is also apparent. This may reflect increased light levels at the forest floor following clearance for the *renvall*. Alternatively, growth of sedges may have been promoted by the creation of small pools on the fen surface through the cutting of peat to fuel the smudge fires. Several open landscape indicators are recorded, such as Chenopodiaceae, Caryophyllaceae, *Epilobium*-type, *Melampyrum* and Rosaceae (Behre 1981; Edwards and MacDonald 1991).

#### First abandonment of the renvall (~1920–1970)

The most obvious changes in AKK D-2 are an increase in the abundance of *Vaccinium* alongside a more continuous presence of *Calluna* and *Empetrum*. Following an initial fall, the PAR of *Picea* rises. *Pinus* and *Betula* increase throughout the zone to levels indicating a local presence. All of these changes are likely to be related to a succession towards boreal forest following abandonment, generally starting with a stage dominated by ericaceous heaths, followed by the rise to dominance of *Betula*, *Pinus* and finally *Picea*. Poaceae do not disappear completely from this LPAZ and traces of *Rumex*-type, *Artemisia* and *Epilobium*-type pollen remain, suggesting that the canopy was not completely closed. Part of the increase in *Pinus* may derive from the introduction of forest management and sustainable forestry, which began in 1906 (Östlund 1995).

The continued but reduced presence of Cyperaceae—most obvious in the PAR diagram (Fig. 6)—could relate to the replacement of sedges by ericaceous heaths within the *renvall* as recovery of the natural forest vegetation progressed, or to the limited creation of new damp habitats due to either the cessation of peat cutting, or the development of a warmer and drier climate during the 20th century (Seppä et al. 2009; St. Amour et al. 2010; Lindholm et al. 2012). C:P is reduced compared to the previous LPAZ, which could reflect the absence of smudge fires. A dip in LOI at AKK D at 8–7 cm (~1969), just below the AKK D-2/3a boundary, may be related to a very brief period of increased soil erosion as the *renvall* was expanded with the addition of the phase 2 annex and hut (Fig. 2; Aronsson 1991).

#### Re-use of the renvall for extensive reindeer herding (~1970–1990)

For AKK D-3a, the similar composition of shrubs (including heaths) and herbs when compared to AKK D-1c (Figs. 5, 6), as well as a rise in *Sporormiella*-type spores and C:P, appears to reflect the reintroduction of reindeer herding. This event is dated to ~1970, and the timing closely fits with the oral history for the site. The abrupt reduction of *Vaccinium*-type pollen to trace values suggests that it may have been severely affected by grazing pressure such as defoliation, trampling and fertilization (Sørensen et al. 2009) within the *renvall*. The relatively small increase in Poaceae and absence of several herbaceous taxa (Caryophyllaceae, *Epilobium*-type and *Melampyrum*) that were recorded during the previous (intensive) stage of use for the pen (AKK D-1c), as well as a discontinuous presence of *Artemisia* and a lack of increase in community diversity, all imply that the impact of reindeer herding during this second, extensive phase was relatively weak. This can perhaps be explained by the change in function for the *renvall—*now used for calf marking rather than milking—which required the herd to be on site for a shorter period (up to 24 h), compared to the several consecutive weeks required for reindeer milking.

#### Recent abandonment (~1990–present)

LPAZ AKK D-3b reflects the most recent phase of abandonment, which the age-depth model estimates to have started around 1990. A recovery of selected trees and shrubs (*Pinus*, *Picea* and *Vaccinium*-type) is visible in the influx data, together with a reduction in Cyperaceae, and an overall decline in community diversity as herbaceous taxa indicative of landscape openness disappear. This explains the central position of pollen assemblages from this LPAZ along axis 1 in the PCA (Fig. 11). The recovery was relatively rapid compared to AKK D-2, probably because the impact of reindeer herding during AKK D-3a was not as intense or sustained as that of the earlier phase. The timing of the palynological signal for abandonment is in good agreement with the observation that the *renvall* was still in use during the 1980s. The elevated total pollen influx values seen in this LPAZ may be explained by the regeneration of trees, notably *Pinus* and, to a lesser extent, *Picea*. There is a reduction in C:P and this would be expected in the absence of smudge fires.

### Comparison of profiles

**Fig. 12:**
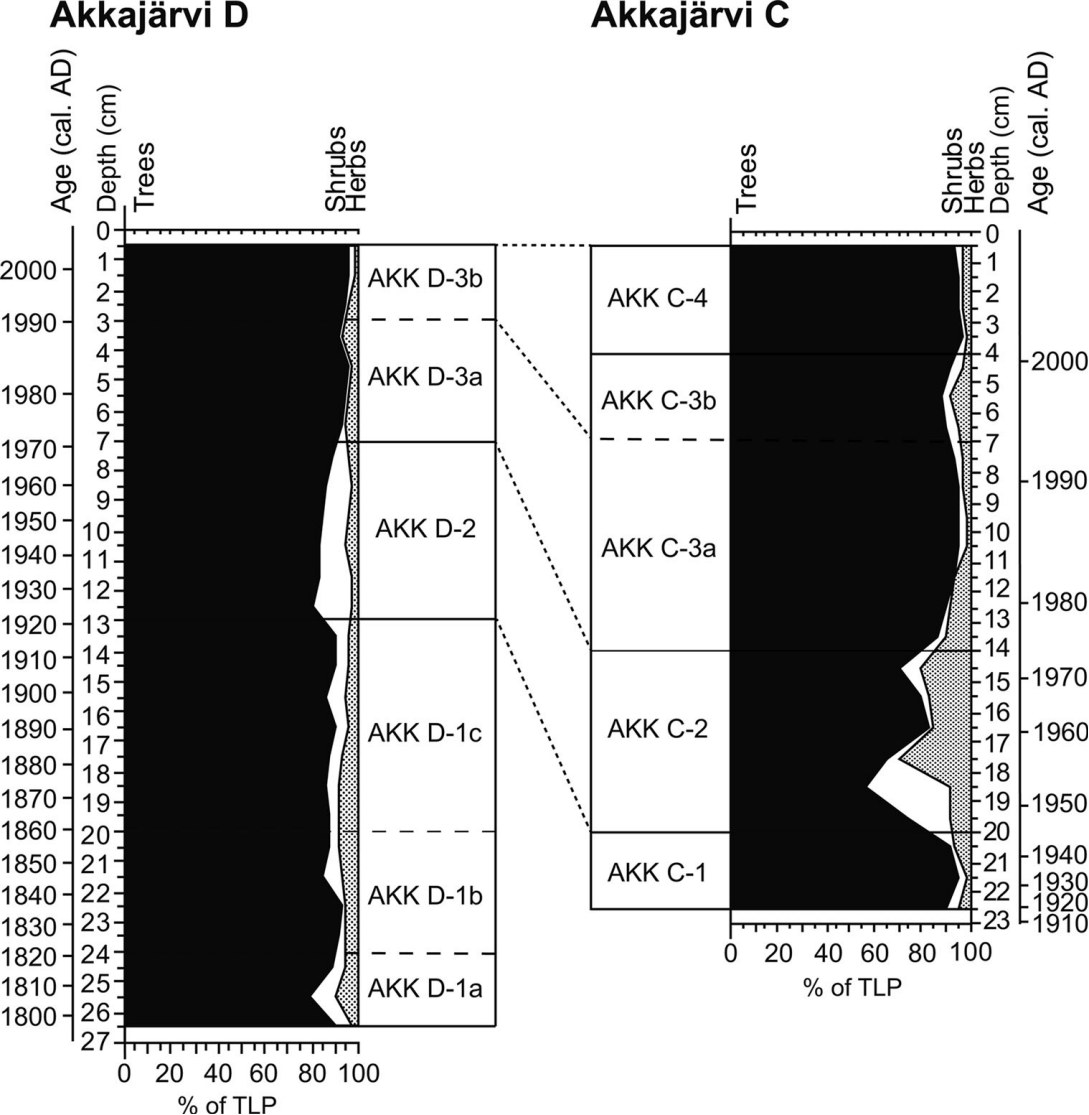
Comparison of the summary pollen diagrams for Akkajärvi D (AKK D) and Akkajärvi C (AKK C). Correlation between the LPAZs is indicated by dashed lines

The correspondence between the LPAZs for AKK C (fen) and AKK D (annex) is summarized in Fig. 12. The latter profile covers a longer period of time, ~200 cal yr compared to 100 years for the fen, with similar vegetational changes displayed at both locations during the period when the cores overlap chronologically. A notable vegetation change recorded in both sequences is the regeneration of heaths, particularly *Vaccinium* (Figs. 5, 6, 9, 10) from the start of the first abandonment phase, beginning ~1920. This seems likely to reflect a recovery of *Vaccinium*-dominated plant communities within the abandoned *renvall*, perhaps with additional contributions of pollen resulting from the local expansion of crowberry over dry hummocks on the fen in response to the warmer and drier climate of the modern period. Herbaceous pollen is minimal throughout both profiles. Pollen of apophytes (taxa that are native but are favoured and spread, directly or indirectly by cultural activity and colonize newly established biotopes; Behre 1988) linked to Sami activity is registered in both profiles, but only at trace values, making the palynological signal for reindeer herding very difficult to distinguish from the background pollen rain produced by the surrounding boreal forest vegetation. This signal weakens with increasing distance from the locus of human and animal activity, as evidenced by the reduced frequency of herbaceous pollen types recorded at AKK C compared to AKK D during the phases when the *renvall* was in use. A stronger signal for herding is providing through changes in microscopic charcoal—which increases in abundance during the periods when the *renvall* was active, probably as a consequence of the lighting of smudge fires—and the coprophilous fungal spore record. The latter is discussed in further detail below.

### Coprophilous fungal spores as indicators of land use

Overall, the palynological records obtained from within the annex and on the fen display broadly similar patterns (Figs. 5, 9) which match chronologically (Fig. 12). In both sequences the herbaceous pollen content is minimal, meaning that the detection of past human impact on the environment in this context depends heavily on the patterns shown by the coprophilous fungal spores within the *renvall* annex (AKK D). A peculiarity arises in that the coprophilous fungal spore signal from the fen (AKK C) is out of phase with that from the *renvall*. High spore frequencies are recorded at AKK D during the two reindeer herding phases (LPAZs AKK D-1c and AKK D-3a). At the same time, percentages of fungal spores are suppressed on the fen (LPAZs AKK C-1 and 3a). Conversely, levels of *Sporormiella*-type (HdV-113) are elevated on the fen during presumed periods of abandonment (LPAZs AKK C-2, -3b and -4) at a time when only traces of these spores are recorded inside the reindeer pen (LPAZs AKK D-2 and D-3b).

This pattern might be explained if the chronologies for the profiles are also slightly offset, yet there is no reason to believe that the age-depth model for either of the two sequences, which is largely based on ^210^Pb dating with very small associated errors, is inaccurate. Both AKK D and AKK C display apparent stratigraphic integrity and the palaeoecological signal for reindeer herding and abandonment at AKK D closely matches the oral history of the site.

The spatial patterns presented in the coprophilous fungal spore data could reflect the use by reindeer of separate areas at Akkajärvi at different times. Taphonomic studies demonstrate that coprophilous fungal spores do not travel far from their source (Raper and Bush 2009), with dispersal often limited to ≤2.5 m from the fruiting body (Ingold 1971; Yafetto et al. 2008). Spores released from fungi growing on dung within the *renvall* (AKK D) might not arrive at AKK C, 15 m from the annex boundary. This would account for the strong fungal spore signature within the reindeer pen, and the weak to absent signal from the fen, during periods when the *renvall* was in use.

More puzzling are the elevated frequencies of fungal spores recorded on the mire (AKK C) and the subdued percentages of spores deposited in the annex (AKK D), during periods when the *renvall* was not in use. The creation of this pattern would require a situation where reindeer regularly returned to graze at the mire but had little, if any, access to the interior of the pen. The mire at Akkajärvi is situated within the wider pasturing grounds for reindeer in this region, yet this raises the additional question as to why the frequencies of coprophilous fungal spores do not remain more constant throughout the mire profile, unless animals were kept away from this area when the pen was in use. Wood and Wilmshurst (2012) argue that the interpretation of records for coprophilous fungal spores (specifically *Sporormiella*-type) from wetlands is far from straightforward, and that spore abundances can fluctuate in response to changes in mire surface hydrology.

It would seem that coprophilous fungal spores are a reliable indicator of reindeer herding activity when studied within a known locus of activity, provided that there is an awareness of the fact that the signal can weaken significantly over short distances from the gathering locations. At AKK D, within the *renvall* annex, the record for coprophilous fungi satisfactorily matches the oral history of Sami activity at the site, while the impacts on the vegetation as revealed through the pollen record appear to be minimal, with the signal from ‘indicator’ taxa arising from disturbance often masked by the pollen from high-pollen producing trees such as *Betula* and *Pinus*. In such cases, the analysis of coprophilous fungi becomes an important means for establishing the local (‘on-site’) presence of herbivores.

Establishing an empirical relationship between herbivores and the abundance of coprophilous fungal spores in the fossil record is proving elusive, but it is generally considered that greater numbers of animals will produce larger quantities of dung and, therefore, higher numbers of spores (Baker et al. 2013). Variations in spore numbers are therefore typically interpreted as representing changes in the size of herbivore populations (Davis 1987; van Geel et al. 2003; Davis and Shafer 2006), with *Sporormiella*-type (HdV-113) considered to be the most useful indicator of herbivore presence (Davis and Shafer 2006; Raper and Bush 2009; Feranec et al. 2011). It should be noted that the characteristically large herds present during the extensive reindeer herding phase at Akkajärvi between 1970 and 1990 resulted in similar frequencies of coprophilous fungal spores being recorded when compared with those during the intensive reindeer herding phase (~1860–1920) with its much smaller-sized herds. The absence or limited presence of Sordariales-type and *Cercophora*-type (HdV-112) spores during the latter phase formed the main difference in the coprophilous fungal spore signature between the two periods of on-site activity (Fig. 5). During the intensive phase, reindeer would be present at the *renvall* for milking purposes over several weeks during the summer months, whereas during the extensive phase it was used for calf marking over a period of a day at most. This could imply, unsurprisingly, that the abundance of certain coprophilous fungi may not only be controlled by herbivore density, but also by the duration of the presence of the animals on the site.

## Conclusions

High resolution palynological analyses, combined with Bayesian age-depth modelling of ^210^Pb and ^14^C dates, has allowed the identification of two multi-decadal periods of *renvall* use in northern Sweden. The first period was dated to ~1860–1920, a time when intensive reindeer herding was still practised, and the second (~1970–1990) followed the introduction of extensive herding practices. The timing obtained for these two phases broadly fits oral histories for the site that suggest abandonment around 1910–1920, followed by re-use around 1960–1970, and a subsequent abandonment during the late 1980s. The impacts of reindeer herding on vegetation across the site appear slight in the pollen record. The signal for activity is characterised by trace values of pollen of *Epilobium*-type and various ruderal plants such as *Rumex*-type, Chenopodiaceae, Caryophyllaceae and *Melampyrum*, and abandonment is indicated by successive increases in Poaceae, ericaceous heaths, particularly *Vaccinium*-type, and finally the trees *Betula* undiff. and later *Pinus* and *Picea*, as the forest reverted back towards a more natural state.

Reindeer herding can be distinguished most clearly in the coprophilous fungal spore record from a peat profile collected inside an annex to the *renvall*. This signal appears to weaken significantly over a short distance (<15 m) from the source of the activity due to the limited capacity for dispersal of these types of spores. Differences in the impact of intensive and extensive reindeer herding are most apparent in the records for coprophilous fungal spores. During intensive reindeer herding, small numbers of animals would have spent several weeks at the *renvall*, resulting in an increase in the abundance and community diversity of coprophilous fungal spores. This is in contrast to large herds spending up to one full day at the site during the extensive reindeer herding period, during which the input of these NPPs is relatively muted. This demonstrates that not just herd size, but the duration of reindeer presence on the site, also influences the abundance of coprophilous fungal spores recorded in the fossil record.

## Electronic supplementary material

Below is the link to the electronic supplementary material.

Supplementary material 1 (PDF 340 kb)
