# Supplementary material for: High-resolution palynology reveals the land use history of a Sami renvall in northern Sweden
Source: Veg Hist Archaeobot. 2016 Nov 18;26(4):369–88. doi: 10.1007/s00334-016-0596-5 (PMC6979733; doi:10.1007/s00334-016-0596-5)

Kamerling, IM, Schofield JE, Edwards KJ, Aronsson K-Å (2016) High-resolution palynology reveals the land-use history of a Sami *renvall* in northern Sweden. *Vegetation History and Archaeobotany* (*corresponding author, ilse.kamerling@abdn.ac.uk, University of Aberdeen, UK)

ESM Fig. 1 Percentage pollen diagram for Akkajärvi D (AKK D) showing trees, shrubs, herbs, aquatics and pteridophytes that were omitted from the diagram displaying selected palynomorphs (Fig. 5). X-axis units are % TLP (total land pollen; minimum sum of 500). Also included are the uncalibrated ^{14}C dates, a calibrated timescale (AD) based on the age-depth model (Fig. 4, panel B), the lithological column for the sequence and the CONISS diagram. Rare types (<1%) are indicated by a + symbol

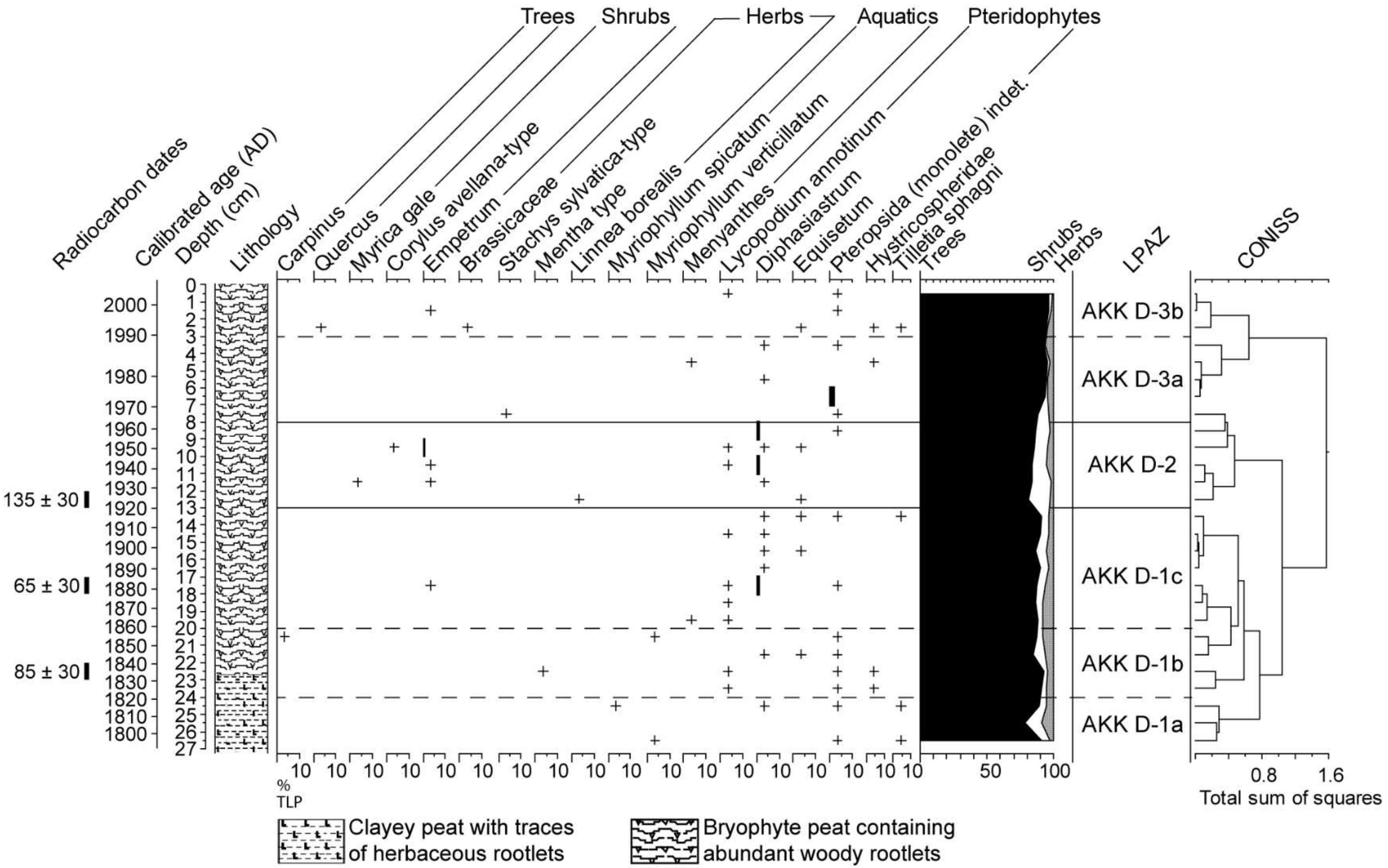

ESM Fig. 2 Percentage pollen diagram for Akkajärvi C (AKK C) showing trees, shrubs, herbs, aquatics and pteridophytes that were omitted from the diagram displaying selected palynomorphs (Fig. 9). X-axis units are % TLP (total land pollen; minimum sum of 500). Also included are the uncalibrated ^{14}C dates, a calibrated timescale (AD) based on the age-depth model (Fig. 8), the lithological column for the sequence and the CONISS diagram. Rare types (<1%) are indicated by a + symbol

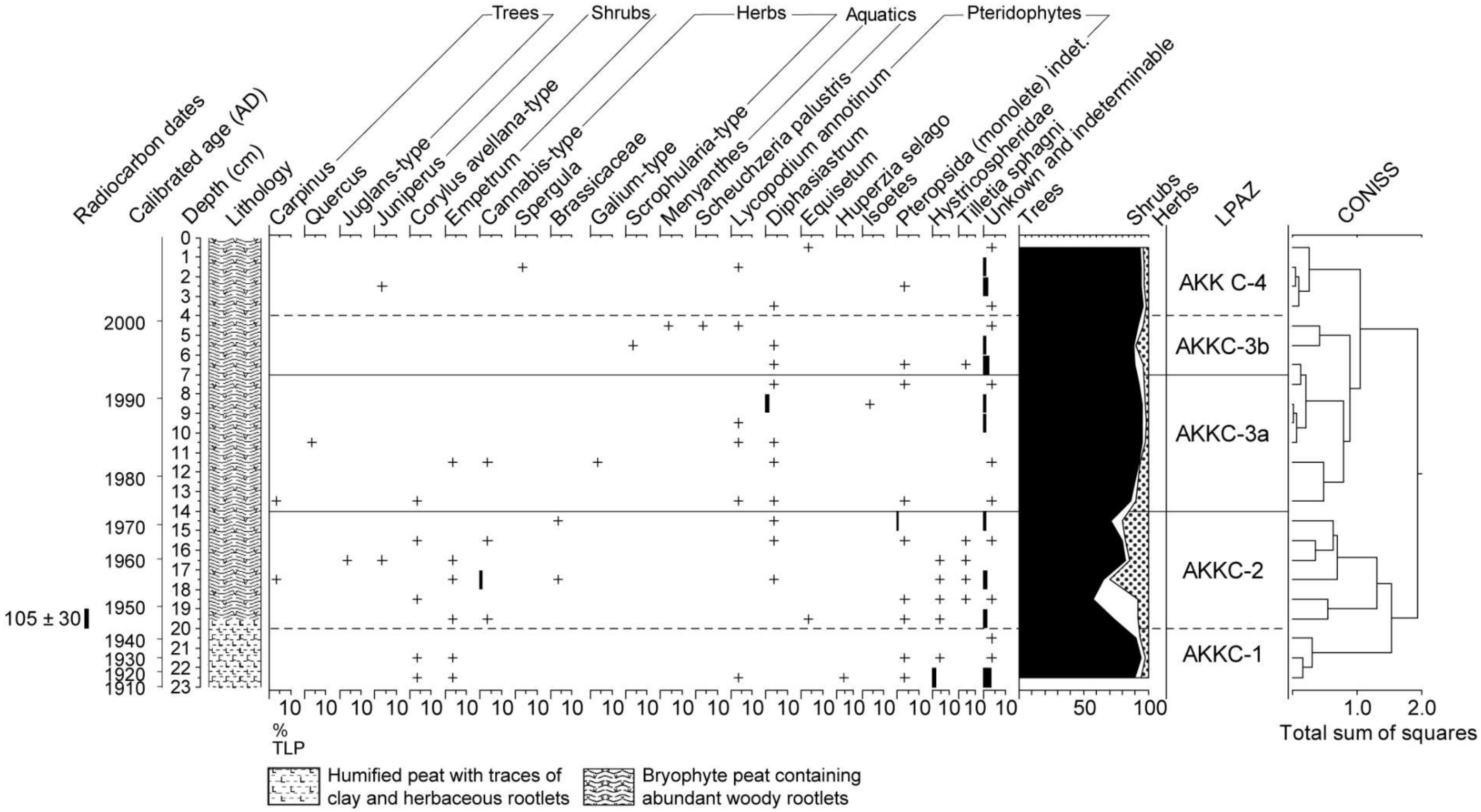

Supplement: Supplementary file 1 — Supplementary material 1 (PDF 340 kb) [file 334_2016_596_MOESM1_ESM.pdf]
